# Engineering *Saccharomyces cerevisiae* for co-utilization of d-galacturonic acid and d-glucose from citrus peel waste

**DOI:** 10.1038/s41467-018-07589-w

**Published:** 2018-11-29

**Authors:** Ryan J. Protzko, Luke N. Latimer, Ze Martinho, Elise de Reus, Tanja Seibert, J. Philipp Benz, John E. Dueber

**Affiliations:** 1 Department of Molecular and Cell Biology, University of California, Berkeley, CA 94720 USA; 2 Department of Chemistry, University of California, Berkeley, CA 94720 USA; 3 Department of Bioengineering, University of California, Berkeley, CA 94720 USA; 4 Department of Biotechnology and Biomedicine, Technical University of Denmark, 2800 Kgs Lyngby, Denmark; 5 Holzforschung München, TUM School of Life Sciences Weihenstephan, Technische Universität München, Freising, Germany; 6 Max Planck Institute of Molecular Plant Physiology, Potsdam, Germany; 7 Biological Systems & Engineering Division, Lawrence Berkeley National Laboratory, Berkeley, CA 94720 USA

## Abstract

Pectin-rich biomasses, such as citrus peel and sugar beet pulp, hold promise as inexpensive feedstocks for microbial fermentations as enzymatic hydrolysis of their component polysaccharides can be accomplished inexpensively to yield high concentrations of fermentable sugars and d-galacturonic acid (d-galUA). In this study, we tackle a number of challenges associated with engineering a microbial strain to convert pectin-rich hydrolysates into commodity and specialty chemicals. First, we engineer d-galUA utilization into yeast, *Saccharomyces cerevisiae*. Second, we identify that the mechanism of d-galUA uptake into yeast is mediated by hexose transporters and that consumption of d-galUA is inhibited by d-glucose. Third, we enable co-utilization of d-galUA and d-glucose by identifying and expressing a heterologous transporter, GatA, from *Aspergillus niger*. Last, we demonstrate the use of this transporter for production of the platform chemical, *meso*-galactaric acid, directly from industrial Navel orange peel waste.

## Abstract (web summary)

Pectin-rich agricultural byproducts are ideal feedstocks for biobased chemicals production. Here, the authors engineer the yeast, *S. cerevisiae*, in several steps to co-utilize d-galacturonic acid and d-glucose and demonstrate the potential of producing *meso*-galactaric acid from industrial orange peel.

## Introduction

Developing technologies for sustainable chemical and energy production represents an important societal challenge. Metabolic engineering is a promising route for producing chemicals in environmentally-friendly fermentations from renewable feedstocks^1,2^. The cost of input feedstocks often limit what commodity chemicals can be produced in an economically-viable manner^3–5^. Furthermore, biofuel production and biorefinery processes from crop plants have faced criticism for demanding valuable resources that overlap with food production, such as water, fertilizer, and arable land^6,7^. Thus, sustainable and cost-efficient feedstocks for the bioproduction of commodity chemicals will be critical for the success of industrial metabolic engineering strategies.

Pectin-rich agricultural byproducts from fruit and vegetable processing possess several qualities of ideal fermentation feedstocks. First, there is ample supply. Annually, citrus fruits for juicing and sugar beets for the refined sugar industry are produced worldwide at 20^8^ and 270 million tons^9^, yielding residues equivalent to 2 and 14 million tons of dry pulp, respectively^10^. Additionally, juicing of the global cash crops, apples, grapes and agave, generate pulpy wastes of high pectin content, representing 20–40% of the dry weight^11–13^. These residues are collected at their processing plants, partially pretreated during sugar and juice extraction, and naturally devoid of lignin—which presents a considerable depolymerization challenge for lignocellulosic feedstocks^14,15^. Despite these advantages, fermentation of pectin-rich agricultural wastes is currently unrealized at commercial scale. Utilizing these byproducts for chemical production would add value without augmenting land use and eliminate the contribution of these agricultural wastes to landfill overflow. While highly esterified pectins from citrus have high-value applications in small food and pharmaceutical markets, the majority of raw material is sold as a livestock feed providing nominal economic returns due to the high cost of drying and pelleting^13^. Furthermore, use of wet material in hydrated fermentations could avoid costs associated with drying. Lastly, the citrus juice and sugar markets have historically exhibited volatility^16^. Conversion of crop byproducts to high-value commodities in isolated markets could de-risk the seasonal variability of crop value.

Pectin is a complex polysaccharide composed of an α-(1,4)-linked d-galacturonic acid (d-galUA) backbone, with this monosaccharide composing over 70% of the polymer^17^. Although pectin offers advantages as a fermentation feedstock, there are technical challenges that must be overcome. Since d-galUA is oxidized compared to neutral hexoses (e.g., galactose or glucose), pectin hydrolysates equilibrate at low pH due to the high concentration of free d-galUA (pKa = 3.5). This acidic environment is favorable for fermenter hygiene by limiting the growth of contaminating microbes, but it limits which production hosts are physiologically viable. Buffering at high pH with titrants eliminates this hygiene benefit and results in byproduct formation (e.g., gypsum). Both filamentous fungi and yeast have been proposed as fermentation hosts for pectin-rich wastes due to their tolerance of acidic conditions and use in industrial processes. Filamentous fungi have been studied for their native d-galUA metabolism, transport, and secretion of pectinases for depolymerization of pectin cell walls^18^. These efforts have culminated in the engineering of an *Aspergillus niger* strain that produced 3.1 g/L *meso*-galactaric acid from citrus peel in a consolidated bioprocess by disruption of d-galUA catabolism and expression of uronate dehydrogenase (UDH)^19^. While use of filamentous fungi for consolidated bioprocessing appears to be an advantage, pectin-rich wastes can be inexpensively and efficiently hydrolyzed by commercial pectinases or acid hydrolysis to yield component monosaccharides^20,21^. Use of *S. cerevisiae* offers several advantages as a fermentation host over filamentous fungi, including superior rapid genetic manipulations for metabolic engineering and production of valued coproducts, such as ethanol^22–24^. Yeast additionally exhibit high growth rates, which reduce fermentation times, and can ferment under anaerobic conditions. These factors lower capital and operating costs while reducing likelihood of contaminating microbes^25^. However, the main limitation of *S. cerevisiae* as a fermentation host for pectin-rich wastes is that it only consumes the fermentable sugars (primarily d-glucose), while d-galUA fractions remain unutilized^21,24^.

Several attempts at engineering *S. cerevisiae* as a host for d-galUA fermentations have been published, yet a strain capable of growing on this sugar as the primary carbon source or co-consuming d-galUA with d-glucose have not yet been reported^24–27^. Efforts to enable growth on d-galUA using a fungal catabolic pathway have been impaired by low specific activity of enzymes within the pathway, particularly the l-galactonate dehydratase step^26,28^. Additionally, *S. cerevisiae* lacks a dedicated transport system for d-galUA and import inhibition of d-galUA by d-glucose has not been described prior to this report. Previous experimental evidence demonstrated uptake of d-galUA into wild-type *S. cerevisiae* occurs near the pKa of d-galUA through an unspecified, channel-type mechanism not inhibited by d-glucose and not dependent on hexose transporters^29^. Surprisingly, we observe that consumption of d-galUA at low pH in engineered *S. cerevisiae* is dependent on yeast hexose transporters and limited in the presence of d-glucose. Studies engineering d-galUA utilization while co-feeding d-galUA and d-glucose likely failed due to this transport inhibition by d-glucose^24^. Uptake inhibition of d-galUA poses a considerable challenge, as pectin-rich hydrolysates contain high concentrations of d-glucose and result in biphasic utilization of d-glucose and d-galUA. This challenge is comparable to the near ubiquitous selective uptake of d-glucose over other monosaccharides in *S. cerevisiae*, including d-galactose and pentoses. Accordingly, co-transport of d-xylose and d-glucose in hemicellulose hydrolysates has remained a heavily investigated area of yeast engineering^30,31^.

In this study, we address these challenges for d-galUA utilization from pectin-rich agricultural feedstocks. We report the development of an engineered *S. cerevisiae* strain capable of growth on d-galUA media. Using this strain, we identify a high-flux transporter capable of d-galUA uptake in low and high pH media and allowing for co-consumption of d-galUA and d-glucose in low pH hydrolysate. Lastly, we demonstrate use of this transporter for direct bioconversion of d-galUA in raw citrus hydrolysate into a renewable chemical, *meso-*galactaric acid, while achieving co-transport and utilization of d-glucose for improved redox balance and coproduction of ethanol. Together, these strain modifications lay the groundwork for using *S. cerevisiae* as a platform host for converting pectin-rich waste streams into specialty and commodity chemicals of industrial interest with high productivity.

## Results

### Engineering *S. cerevisiae* for utilization of d-galUA

A metabolic pathway endogenous to filamentous fungi for converting d-galacturonic acid to the central carbon metabolite pyruvate and glycerol was originally described in *Aspergillus niger*^18^. We hypothesized that in vivo production of pyruvate and glycerol from d-galUA would confer growth of *S. cerevisiae* on d-galUA as the sole carbon source. We first sought to isolate enzymes with high activity for each pathway step by overexpressing and assaying enzyme activity in yeast cell lysate. First, we constitutively expressed codon-optimized individual genes from various fungi in laboratory *S. cerevisiae* strain BY4741 and assayed for the respective enzyme activity in lysate (Supplementary Table 1; Supplementary Data 1). We found that d-galacturonate reductase from *Trichoderma reesei* (GAR1) and 2-keto-3-deoxy-L-galactonate aldolase from *Aspergillus niger* (GAAC) have activities near the rate-limiting specific activity of glycolysis: 0.1 µmol min^−1^ mg^−1^ protein (Supplementary Table 2)^32^. However, consistent with previous reports^26,28^, we observed very low activity for the three L-galactonate dehydratase (LGD1) enzymes tested. To allow for facile detection of dehydratase expression, we N-terminally tagged the *T. reesei* LGD1 with a yellow fluorescent protein (Venus). Fortuitously, this fusion increased dehydratase activity in lysate 60-fold. To determine if the Venus fusion improved protein expression, stability or solubility, we expressed C-terminally Flag-epitope tagged LGD1, Venus-LGD1, and MBP-LGD1 (maltose binding protein; a common solubilization domain) to enable protein lysate analysis by western blot (Supplementary Fig. 1). We observed no qualitative difference in solubility or total amount of protein between all three samples. The l-galactonate dehydratase activity for the MBP-LGD1 was 5.4-fold higher than untagged LGD1 (Supplementary Table 2). Additionally, the *K*_m_ of the Venus-LGD1 was identical to that of the purified wild-type LGD1 (Supplementary Table 2).

**Fig. 1:**
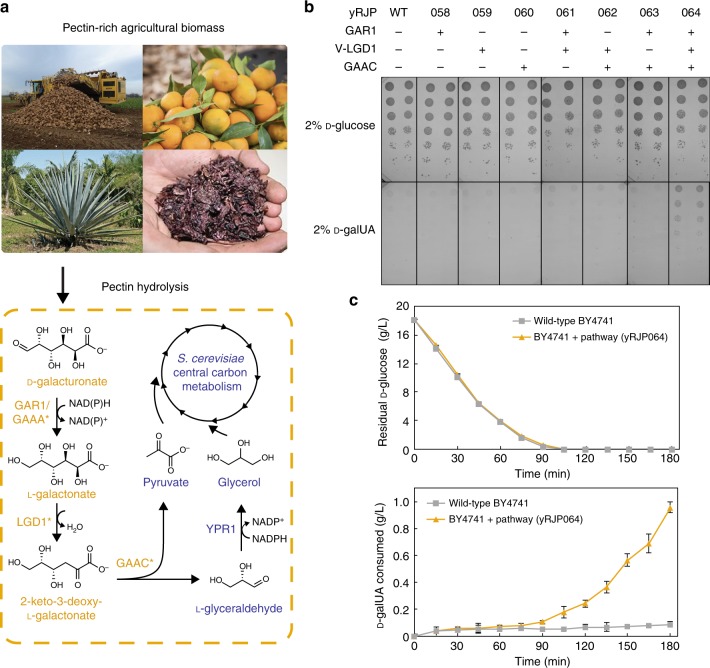
Utilization of d-galacturonic acid by engineered *S. cerevisiae*. **a** Pectin-rich agricultural byproducts include sugar beet pulp, citrus peels, agave pulp and grape pomace. Hydrolysis of pectin yields d-galacturonic acid (d-galUA) monomers. The d-galUA catabolic pathway from filamentous fungi (orange asterisks) can be heterologously expressed in *S. cerevisiae* to yield intermediates in native metabolism (blue). **b** Expression of high activity GAR1, Venus-LGD1, and GAAC are necessary and sufficient for *S. cerevisiae* growth on d-galUA minimal media plates. Serial spotting of *S. cerevisiae* strains expressing all combinations of d-galUA catabolic enzymes on D-galUA minimal media plate at pH = 3.5. **c** Wild-type (gray squares) and yRJP064 (orange triangles) cultures were incubated in pH 3.5 orange peel hydrolysate media at high density (OD_600_ = 75) and concentrations of d-galUA and d-glucose were monitored by HPLC. Consumption of d-galUA (14.1 g/L starting concentration) by yRJP064 coincides with depletion of d-glucose in media. Images sourced from Stan Shebs, Adrian J. Hunter, and 4028mdk09 Wikimedia Commons, under a Creative Commons Attribution 3.0 license and Betsisman under Creative Commons CC0 license

Previous research has shown that near pH 3.5, the pKa of d-galUA, this uronic acid is rapidly imported from the media into *S. cerevisiae* by an unknown mechanism^29^. To assay for growth, we took advantage of this import mechanism by growing on agar plates with d-galUA as the sole carbon source buffered at pH 3.5. We expressed multigene expression cassette plasmids with all combinations of the utilization pathway enzymes to determine which steps were necessary for growth (Fig. 1). We found a strain (yRJP064) expressing GAR1, Venus-LGD1, and GAAC formed visible colonies, while the wild-type strain or incomplete pathways (yRJP058-063) were incapable of promoting growth. Having identified the minimal necessary enzymatic steps for growth in d-galUA media, we sought to improve growth by designing a combinatorial library containing multiple expression levels of enzymes, cofactor usages, and inclusion of fungal l-glyceraldehyde reductases (Supplementary Fig. 2A; Supplementary Table 4; Supplementary Methods). We transformed this library into BY4741 and found that after three rounds of enrichment in d-galUA liquid media, the doubling times of the culture and final ODs stabilized (Supplementary Fig. 2B). We sequenced an enriched strain (yRJP221) and found it contained a plasmid (pRJP1444) with the d-galUA reductase (GAAA) from *Aspergillus niger*, Venus-LGD1 and GAAC at high expression levels (*pTDH3*). Interestingly, despite the lower specific activities measured for GAAA compared to GAR1 in cell lysate (Supplementary Table 2), the growth enrichment selected for GAAA which is capable of utilizing both NADPH and NADH for reducing d-galUA, whereas GAR1 strictly uses NADPH^33^. The sequencing identified no mutations in pRJP1444, so to determine if this plasmid was sufficient for improved d-galUA utilization, we retransformed it into our original BY4741 strain, yielding strain yRJP222. We observed identical growth profiles for yRJP221 and yRJP222 on d-galUA, indicating that the plasmid, rather than genomic mutations, was responsible for the growth on d-galUA.

Glycerol accumulated in our engineered strains during incubation with d-galUA (Supplementary Fig. 3B), likely due to the poor utilization of glycerol by BY4741. Suspecting slow glycerol utilization to be limiting to growth, we transferred the pRJP1444 plasmid into a strain optimized for glycerol utilization (CEN.PK113-1A-GUT1_JL1_UBR2_CBS_^34^), yielding strain yRJP224, and assayed for growth on d-galUA media. Excitingly, we observed higher saturating densities for yRJP224 in d-galUA media compared to our precursor strains and yRJP223, a wild-type CEN.PK strain expressing the catabolic pathway (Supplementary Fig. 2C). To assay the utilization of d-galUA and resulting glycerol by these strains on pectin-rich agricultural waste, we received Naval orange peels as a gift from an industrial juicing facility and prepared CPW hydrolysate as previously described^21^. We assayed growth of strains yRJP223 and yRJP224 in CPW hydrolysate media starting from low starting densities (OD_600_ = 0.1) over 90 h. We observed d-galUA consumption for both strains (3.0 ± 0.2 g L^−1^ d-galUA and 5.1 ± 0.3 g L^−1^ for yRJP223 and yRJP224, respectively) and growth during the d-galUA utilization phase after glucose was depleted (Supplementary Fig. 4). We found that the glycerol utilization strain, yRJP224, did not accumulate a final glycerol titer and co-consumed glycerol with d-galUA, whereas the wild-type CEN.PK strain, yRJP223, accumulated 4.1 ± 0.3 g L^−1^ glycerol.

### Biphasic utilization of d-glucose and d-galUA

During growth of our engineered *S. cerevisiae* strains in acidic CPW hydrolysate, we observed minimal consumption of d-galUA (0.2 ± 0.1 g L^−1^ and 0.1 ± 0.2 g L^−1^ for yRJP223 and yRJP224, respectively) until d-glucose was consumed after 21 h (Supplementary Fig. 4). To confirm this biphasic consumption pattern for the two monosaccharides, we incubated high density (OD_600_ = 75) cultures of our engineered BY4741 d-galUA catabolic strain (yRJP064) in CPW hydrolysate and monitored consumption of d-glucose and d-galUA (Fig. 1c). We again observed limited uptake of d-galUA until d-glucose was fully consumed at 90 min. Despite the previously reported co-uptake of d-glucose and d-galUA by biochemical assays^29^, this observation of biphasic consumption of d-glucose before d-galUA suggests that native yeast uptake mechanisms for d-galUA are not sufficient for co-utilization with d-glucose and poses a considerable problem for efficient utilization of d-galUA in pectin-rich hydrolysates. We hypothesized that expression of a specific, high-flux d-galUA transporter in yeast would be required for co-consumption of d-glucose and d-galUA.

### Identification of a high-flux d-galUA transporter

**Fig. 2:**
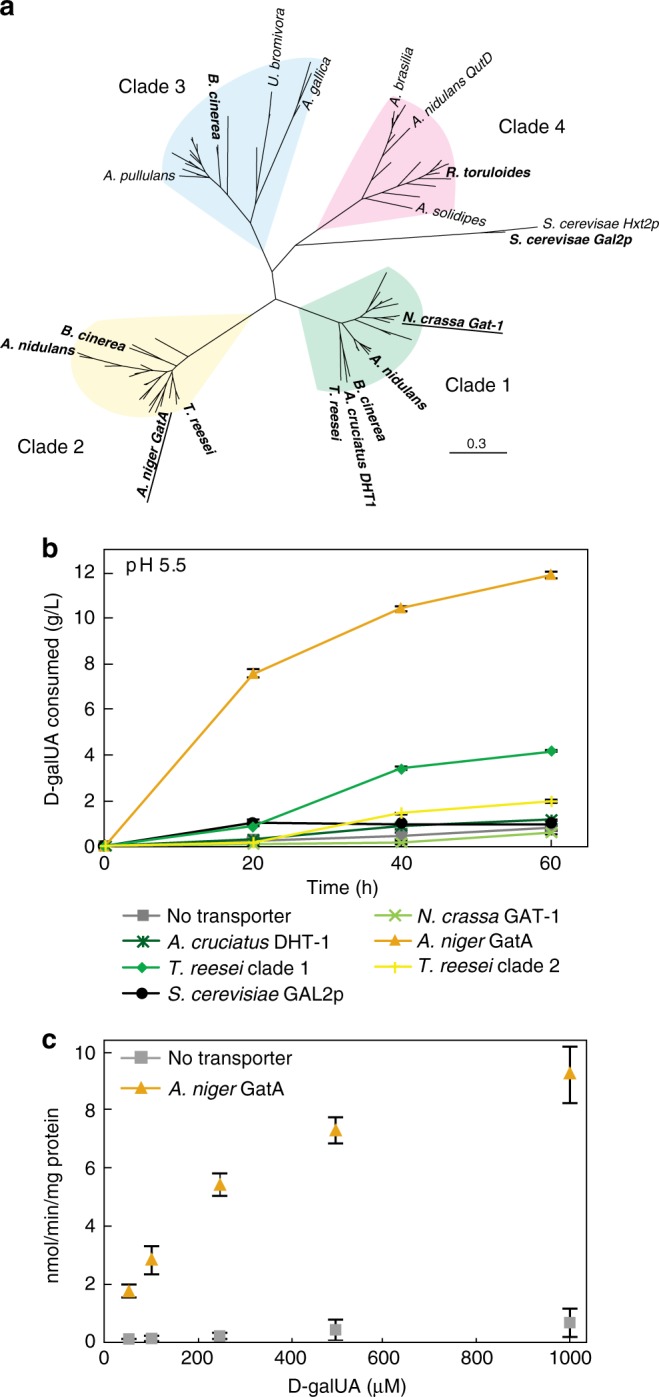
Heterologous transporters enable uptake and utilization of d-galUA by *S. cerevisiae* at high pH. **a** Homologues of the eukaryotic d-galacturonic acid transporter, GAT-1, include members of the quinate transporter family (QutD). **b** Transporters (bolded in A) were expressed in the catabolic strain, yRJP064, and assayed for d-galUA transport and consumption at pH 5.5 (initial OD_600_ = 10). **c** A strain expressing GatA from *Aspergillus niger* exhibits high-flux d-galUA transport in tritiated d-galUA uptake assays with a *K*_m_ of 340 μM and *V*_max_ of 12.1 nmol min ^−1^ mg^−1^ protein

While we previously described the heterologous d-galUA transporter GAT-1 from *Neurospora crassa* as a bona fide d-galUA transporter with activity in *S. cerevisiae*, GAT-1 demonstrates high-affinity, low-flux transport kinetics^23^. We hypothesized that homologs of GAT-1 from various fungi (Fig. 2a) would have diverse kinetic properties and could allow for a more rapid uptake of d-galUA in the presence of d-glucose. As an initial screen for functional transporter expression, we C-terminally tagged GAT-1 transporter homologs with a red fluorescent protein (mRuby) for yeast expression or with GFP for complementation trials of the Δ*gat-1* strain in *N. crassa*. Clades 1 and 2 contain known or putative fungal d-galUA transporters^23,35,36^ and we sampled five and four GAT-1 homologs from these groups, respectively (Supplementary Table 5; Supplementary Data 1; Supplementary Methods). Clades 3 and 4 contain known or putative quinate family transporters (QutD)^37^ and two homologs from these clades were assayed from organisms exhibiting pectin catabolism phenotypes^38,39^. We observed plasma membrane trafficking in *S. cerevisiae* from the *T. reesei* Clade 1 homolog and *A. niger* and *T. reesei* Clade 2 homologs (Supplementary Fig. 5) and at least partial complementation of the Δ*gat-1* phenotype in *N. crassa* by these homologs (Supplementary Fig. 6). To assay transport activity, we integrated untagged transporters into our original d-galUA catabolic strain (yRJP064) and incubated these cultures (OD_600_ = 10.0) in synthetic complete media with d-galUA at pH 5.5, such that uronic acid transport by native mechanisms was restricted and d-galUA consumption could only be achieved by expression of a functional transporter. To control for possible toxicity of these transporters, we additionally incubated cultures at pH 3.5 (Supplementary Fig. 7). We identified An14g04280, or GatA^36^, from *A. niger* as a transporter that allowed for 11.9 g/L consumption of d-galUA at high pH in our catabolic strain during the course of the assay (Fig. 2b), whereas strains expressing no transporter and inactive d-galUA transporters showed negligible consumption. We assayed the kinetics of GatA with tritium-labeled d-galUA and measured a *K*_m_ of 340 μM and *V*_max_ of 12.1 nmol min^−1^ mg^−1^ protein (Fig. 2c). This is an approximately 50-fold increase in *V*_max_ compared to the previously reported GAT-1 expressing strain, which has a *K*_m_ of 1 μM and *V*_max_ of 0.256 nmol min^−1^ mg^−1^ protein^23^.

### Engineering co-consumption of d-galUA and d-glucose

**Fig. 3:**
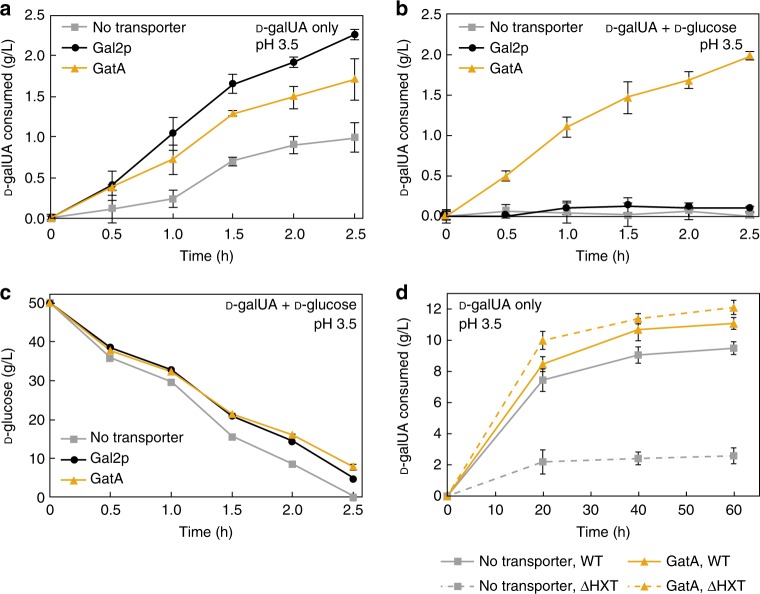
Expression of GatA enables co-consumption of d-glucose and d-galUA. d-galUA utilization strains expressing no transporter (yRJP064), Gal2p (yRJP054) or GatA (yRJP093) were incubated in pH 3.5 media containing 20 g/L d-galUA (**a**) as the sole carbon source or **b**, **c** d-galUA and d-glucose at high density (OD_600_ = 75). **d** Hexose transporters are necessary for consumption of d-galUA at low pH and GatA rescues consumption of a hexose knockout strain. Wild-type and hexose knockout (HXT1-7, GAL2) d-galUA utilization strains expressing no transporter (yRJP193, yRJP195) or GatA (yRJP194, yRJP196) were incubated in pH 3.5 media containing 20 g/L d-galUA (OD_600_ = 10). Error bars represent standard deviation of biological triplicates

As stated previously, hydrolysates of pectin-rich biomass contain high concentrations of both d-galUA and d-glucose^21^. After identifying that consumption of d-galUA was limited by the presence of d-glucose in our engineered catabolic strain using native transport mechanisms in CPW hydrolysate, we sought to assay the co-consumption of d-galUA and d-glucose in the catabolic strain, yRJP064, expressing the high activity *A. niger* d-galUA transporter, GatA. We additionally assayed the constitutive expression of yeast galactose transporter Gal2p, which is reported to have an extremely broad substrate range, including d-xylose, l-arabinose, d-fucose, d-glucose, d-fructose, d-mannose, and d-ribose^40,41^. We incubated high density cultures (OD_600_ = 75) of yRJP064 alone, yRJP064 expressing Gal2p (yRJP054), and yRJP064 expressing GatA (yRJP093), in acidic media containing only d-galUA or both d-galUA and d-glucose. We again observed that d-galUA is consumed at low pH in the absence of d-glucose and that expression of either transporter improves d-galUA consumption (Fig. 3a). This suggests that Gal2p can mediate d-galUA uptake at low pH. However, when d-glucose was present in the media we observed complete inhibition of d-galUA consumption in the catabolic strain without a transporter, as well as when Gal2p was overexpressed (Fig. 3b). Strikingly, the catabolic strain co-expressing the GatA transporter (yRJP093) consumed d-galUA in the presence of d-glucose at a rate similar to that of d-galUA consumption in the media without d-glucose (Fig. 3a, b). d-glucose was present during the entirety of the uptake assay and d-glucose consumption rates were comparable across all strains (Fig. 3c). Thus, heterologous expression of GatA should enable import of d-galUA from raw pectin hydrolysates.

Our observation that d-glucose inhibited d-galUA consumption in wild-type *S. cerevisiae* strongly suggested competing mechanisms of transport between these sugars via hexose transporters. To investigate the involvement of hexose transporters in the wild-type uptake of d-galUA in acidic media, we introduced the fungal catabolic pathway into a strain, KY73, unable to import d-glucose due to knockouts of HXT1-7 and GAL2 yielding yRJP195^42^ and assayed this strain for uptake of d-galUA at pH 3.5. The partial hexose knockout strain exhibited over fourfold lower total consumption of d-galUA compared to the wild-type strain containing a full set of native hexose transporters (yRJP193) (Fig. 3d). The expression of GatA in this partial hexose knockout strain (yRJP196) rescued the consumption d-galUA. Thus, d-galUA must compete with d-glucose for import via hexose transporters unless a d-galUA-specific transporter, such as GatA, is heterologously expressed. Interestingly, while expression of GatA rescued d-galUA transport in the partial hexose knockout strain, we did not observe rescue of growth on d-glucose (Supplementary Fig 8), suggesting that GatA has specificity for d-galUA transport and does not transport sufficient d-glucose to support growth.

Previous tritiated d-galUA uptake assays observed wild-type levels of d-galUA uptake in a hexose knockout strain and did not observe inhibition of d-galUA uptake at low pH by d-glucose^29^. We repeated this assay and observed both rapid association of d-galUA with yeast cells at low pH and that this effect was uninhibited by d-glucose (Supplementary Fig. 9). The biochemical explanation for this discrepancy between the clear inhibition of d-galUA uptake in our consumption assays and the tritiated uptake assay results is yet to be determined.

### Conversion of d-galUA from CPW into *meso*-galactaric acid

**Fig. 4:**
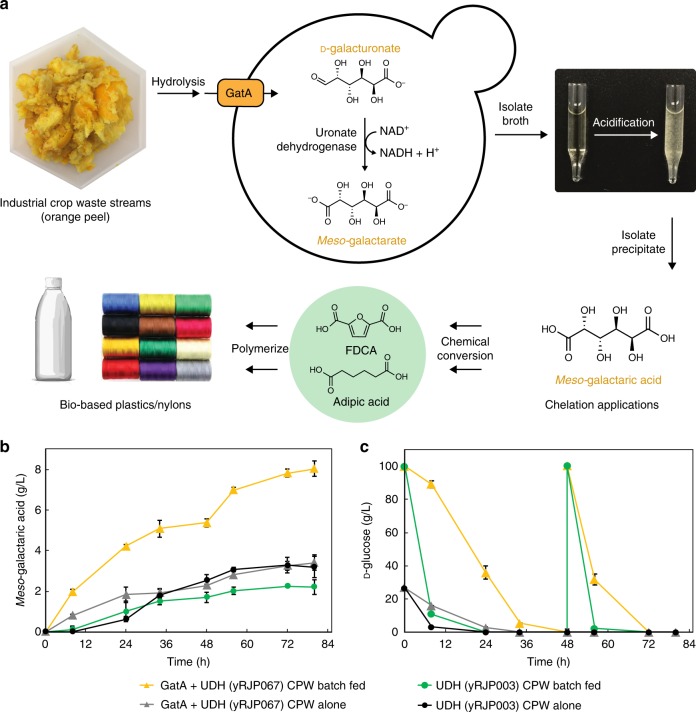
Production of *meso*-galactaric acid from citrus peel by engineered *S. cerevisiae*. **a** Citrus peel is enzymatically depolymerized to yield an acidic hydrolysate containing d-galUA and d-glucose. GatA transports d-galUA intracellularly in the presence of d-glucose. UDH oxidizes d-galUA to *meso-*galactarate, which accumulates in the media and is precipitated by acidification of the broth. *Meso-*galactarate is used directly as a renewable chelate and pharmaceutical conjugate. Efficient chemical conversions of *meso*-galactaric acid yield nylon and plastic monomers, adipic acid and 2,5-furandicarboxylic acid (FDCA), for polymerization and broad commercial applications. **b** Strains co-expressing UDH and GatA (yRJP067, triangles) exhibit higher conversion of d-galUA to *meso-*galactaric acid in acidic orange peel hydrolysate media in the presence of glucose compared to strains expressing only UDH (yRJP003, circles). Batch feeding of d-glucose (**c**) at 0 and 48 h in CPW hydrolysate cultures increases *meso-*galactaric acid yields when expressing GatA (orange triangles), but inhibits yields without the transporter (green circles). Concentrations are normalized for glucose batch feeding. Error bars represent standard deviation of biological triplicates. Image sourced from Petr Kratochvil and licensed under CC0 1.0 Universal Public Domain Dedication by author

Having demonstrated co-consumption of d-glucose and d-galUA in defined medium by expression of GatA, we applied this transporter strategy toward the direct production of a useful specialty chemical, *meso*-galactaric acid, from CPW hydrolysate, as outlined in Fig. 4a. As a platform chemical with applications as a biodegradable chelate, cosmetic, pharmaceutical conjugate, and a biopolymer precursor, *meso-*galactaric acid is a promising target for bioconversion of pectin-rich wastes^43^. In our previous study, we showed that yeast expressing uronate dehydrogenase (UDH) can oxidize d-galUA into *meso*-galactaric acid at µg/L yields^23^. Since both d-glucose and d-galUA are present in high quantities in CPW hydrolysate, we hypothesized that expression of the GatA transporter will allow conversion of d-galUA to *meso*-galactaric acid via UDH even in the presence of d-glucose, which should provide ATP production and redox balancing via glycolysis to achieve higher productivities. Conversely, a strain expressing only UDH should show limited *meso*-galactaric acid production in the presence of d-glucose.

We inoculated CPW hydrolysate alone (measured d-glucose concentration 26.8 g L^−1^) or supplemented with additional d-glucose (starting concentration of 100 g L^−1^) with *S. cerevisiae* cultures (initial OD_600_ = 5.0) expressing either only UDH genomically integrated (yRJP003) or UDH and GatA genomically integrated (yRJP067) (Fig. 4b, c; Supplementary Table 6). In the d-glucose supplemented cultures, once the initial d-glucose was depleted, we added an additional 100 g L^−1^ d-glucose to promote continued fermentation at 48 h. At 8 h of fermentation, while glucose was present in all conditions, the conversion of d-galUA to *meso-*galactaric acid was observed in the UDH and GatA co-expression strain to be 0.12 ± 0.01 and 0.25 ± 0.02 g L^−1^ h^−1^ with hydrolysate alone or glucose added, respectively. In the absence of GatA expression, no *meso*-galactaric product was observed at these time points as predicted by the expected lack of d-galUA import in the presence of d-glucose. After the d-glucose consumption phase in unsupplemented hydrolysate, further incubation of the UDH strains with or without GatA led to production of similar final concentrations of *meso-*galactaric acid. Excitingly, we found that supplementation of d-glucose increased *meso-*galactaric acid production in the UDH strain expressing the GatA transporter, which reached titers of 8.0 ± 0.6 g L^−1^ in 80 h compared to the same strain that was not supplemented with d-glucose, which reached 3.2 ± 0.1 g L^−1^ in 80 h. In d-glucose-fed conditions, a strain expressing only UDH showed a lower titer of 2.2 ± 0.4 g L^−1^, likely due to the inhibition of d-galUA uptake by d-glucose. For both d-glucose-fed strains, we observed ethanol accumulation as a coproduct from d-glucose consumption (Supplementary Fig. 10B). We measured higher relative ATP ratios in the GatA and UDH co-expression strain cultured in the presence of d-galUA and d-glucose (100% ± 2) compared to d-galUA alone (25% ± 1). Furthermore, the presence of d-glucose in the culture increased the NAD^+^/NADH ratio (1.5 ± 0.1) compared to d-galUA alone (0.9 ± 0.1) (Supplementary Table 7; Supplementary Methods). This suggests the role of d-glucose in maintaining higher concentrations of ATP and enabling NADH oxidation, thereby regenerating NAD^+^ for *meso-*galactaric acid production. To further investigate the role of d-glucose in balancing the NADH produced from UDH activity with NADH consumed by glycerol formation, we constructed a set of strains expressing GatA and increasing expression of UDH. We fermented these strains in d-glucose and d-galUA media and measured final *meso-*galactaric acid and glycerol media concentrations. We found that as UDH expression increased, both *meso-*galactaric acid and glycerol increased, indicating that d-glucose provided a redox sink for NADH production through glycerol formation (Supplementary Fig. 11, Supplementary Table 8). We additionally observed ethanol accumulation in our d-glucose-fed hydrolysate conditions as a coproduct from d-glucose consumption (Supplementary Fig. 10B).

## Discussion

Vast global economic and environmental resources are directed toward the growth, harvesting, and processing of pectin-rich crops, including citrus fruits, apples, grapes and agave for juicing^8^, and sugar beets, a top 10 world crop by mass, for sugar production^9^. Large quantities of peel and pulp are already consolidated at processing facilities, partially treated, and more easily hydrolyzed than lignocellulosic material, making them promising feedstocks for fermentations. Furthermore, they offer the potential to produce products with dramatically reduced greenhouse gas emissions compared to existing bioprocesses, such as corn-ethanol, where roughly half the greenhouse gas emissions occur during crop management and collection^44^. In this work, we provide tools enabling *Saccharomyces cerevisiae* to be used as a platform host for the utilization and bioconversion of primary sugars in pectin-waste hydrolysate.

We have identified several areas of yeast strain engineering that warrant future investigation and may further improve catabolism of the d-galUA in these pectin-rich feedstocks. First, we identified the preference for a d-galUA reductase capable of NADH cofactor utilization for growth on d-galUA as the sole carbon source (Supplementary Fig. 2). This suggests balancing d-galUA catabolism with host redox requirements may be a vital design feature. Secondly, we showed that inclusion of the d-galUA utilization pathway in an engineered glycerol-utilizing strain improved growth on d-galUA media and led to consumption of glycerol produced by the heterologous fungal catabolic pathway (Supplementary Fig. 4). Transient accumulation of glycerol in this strain during growth on CPW media suggests further improvements in glycerol catabolism will likely improve growth and d-galUA utilization. Third, flux was improved by the fortuitous 60-fold improvement in l-galactonate dehydratase activity we achieved by tagging the LGD1 enzyme with the Venus fluorescent protein. Uncovering the mechanism for this improvement may aid in expression of other dehydratases and further engineering of these enzymes.

The ability to co-utilize d-galUA and d-glucose by expression of a d-galUA-specific transporter will be necessary for economically-viable fermentations of pectin-rich wastes to maintain productivities in the presence of d-glucose. Sugar co-utilization has been a long-standing problem for yeast fermentations of numerous complex feedstocks, most notably lignocellulosics containing d-glucose and d-xylose^30,31,45^. As evidence of this importance, we observed a biphasic pattern of d-galUA utilization and conversion to *meso-*galactaric acid in strains lacking heterologous expression of the GatA transporter in the presence of d-glucose (Fig. 4). Furthermore, co-import of d-glucose along with d-galUA can maintain the cell in a productive metabolic state. Heterologous expression of GatA and D-glucose co-feeding increased ATP and raised NAD^+^/NADH levels (Supplementary Table 7). A low NAD^+^/NADH ratio in the absence of d-glucose is expected from the activity of UDH, which should deplete intracellular NAD^+^ and produce NADH. The addition of d-glucose, however, provides substrate to re-oxidize NADH, primarily by forming the reduced product glycerol. Indeed, higher glycerol concentrations were observed in glucose-fed GatA/UDH strains compared to UDH alone, and glycerol yields positively correlated with *meso-*galactaric acid titers (Supplementary Fig. 11) suggesting coupling between UDH and glycerol-3-phosphate dehydrogenase in the glycerol formation pathway. Similar findings of increased glycerol formation have been reported in engineered yeast strains overexpressing formate dehydrogenase during co-metabolism of d-glucose and oxidation of formate to carbon dioxide^46^. Future opportunities include coupling d-galUA oxidation by UDH to the production of other reduced coproducts, such as alcohols and polyols.

Use of GatA for co-import of d-galUA and d-glucose has broader implications for the use of d-galUA in redox balancing applications with imbalanced d-glucose metabolic pathways. As first proposed by Van Maris et al.^47^, a heterologous reductive pathway converting d-galUA to central carbon metabolites in *S. cerevisiae* could be used to balance overproduction of NAD(P)H. For example, NADH generated by assimilatory metabolism during anaerobic fermentations of d-glucose to ethanol are naturally balanced by the NADH-consuming production of glycerol, resulting in a lower ethanol yield. The use of d-galUA as an external electron acceptor in engineered *S. cerevisiae* could offset this glycerol production and improve ethanol yields.

In addition to utilization and use in redox balancing applications, d-galUA is a unique starting substrate for bioconversions for production of commercially relevant aldaric acids^48^, aldonic acids^49^, and ascorbic acid (vitamin C)^50–52^. Aldaric acids, *meso*-galactaric acid and d-glucaric acid, have garnered considerable academic and industrial attention for their use as chelates in a broad range of markets, including cosmetics and pharmaceuticals^43,53–55^. This earned d-glucaric acid status as a top target molecule from biomass by the Department of Energy in 2004^56^. Both aldarics offer more effective chelation compared to existing bio-derived chelates, such as citrate^57,58^, and are promising substrates for production of polyhydroxypolyamides (hydroxylated nylons), which have applications as biodegradable polymers, films and adhesives^58–61^. These applications position *meso*-galactaric acid to be commercially relevant. *Meso*-galactaric acid currently sells for up to 100 USD per kg^62^ in cosmetics markets and, given its favorable chelation properties, has the potential to sell at prices comparable to higher volume, biobased chelates such as methylglycindiacetic acid (MGDA, 3–5 USD per kg)^63^. The downstream processing of *meso*-galactaric acid is especially attractive, as it can be precipitated from the fermentation broth by acidification (Fig. 4a)^48,64^. Simple and high purity isolation of *meso*-galactaric acid may enable effective one-pot chemical conversions to the drop-in plastic resin and nylon-6,6 monomers, 2,5-furandicarboxylic acid (FDCA) and adipic acid, respectively^65,66^. FDCA-based poly(ethylene furonate) (PEF) is currently being pursued as a drop-in replacement for conventional, petroleum-based poly(ethylene terephthalate) (PET) plastic^67^ and demonstrates superior gas barrier and mechanical properties^68^. Excitingly, scaling of *meso*-galactaric production from citrus peel waste could allow for production of citrus-peel based PEF plastic bottles for citrus juice packaging and replace the reliance on petroleum-based plastics.

The *S. cerevisiae* technologies described here provide platforms for diverting carbon in pectin-rich peel and pulp wastes globally toward diverse renewable chemical and consumer product markets. Such repurposing of current waste streams has the potential to create rural jobs, de-risk the historically volatile juice and sugar markets by accessing more isolated markets, and concurrently provide sustainable feedstocks for fermentation.

## Methods

### Strains and growth conditions

Single gene expression plasmids were transformed into chemically competent TG1 *E. coli* and multigene plasmids were transformed into TransforMax EPI300 (Epicenter) electrocompetent *E. coli*. Selections were performed on LB containing kanamycin (25 mg L^−1^). *S. cerevisiae* strain BY4741 (MATa his3Δ1 leu2Δ0 met15Δ0 ura3Δ0) was used for experiments in this study and propagated at 30 °C. Yeast strain KY73 (hxt1∆::HIS3::∆hxt4 hxt5::LEU2 hxt2∆::HIS3 hxt3∆::LEU2::hxt6 hxt7::HIS3 gal2∆::DR* ura3–52 his3–11,15 leu2–3,112 MAL2 SUC2 GAL MEL) was used for hexose knockout studies and propagated in YPM (10 g L^−1^ Bacto Yeast Extract; 20 g L^−1^ Bacto Peptone; 20 g L^−1^ maltose). Wild-type yeast cultures were grown in YPD (10 g L^−1^ Bacto Yeast Extract; 20 g L^−1^ Bacto Peptone; 20 g L^−1^ d-glucose). Lithium acetate transformation method^69^ was used to transform yeast with plasmids containing the respective auxotrophic markers. Selection was performed on synthetic dropout media (6.7 g L^−1^ Difco yeast nitrogen base w/o amino acids; 2 g L^−1^ synthetic defined amino acid mix minus the respective autotrophy, w/o yeast nitrogen base (US Biological); 20 g L^−1^ d-glucose or the respective carbon source; 20 g L^−1^ BD Difco agar was used for plates). pH was adjusted when appropriate with NaOH or HCl. A value of 0.21 mg OD^−1^ mL^−1^ +/− 0.02 dry cell weight was calculated for triplicate cultures of BY4741 grown to mid-log in d-glucose synthetic complete media. Navel orange peel from fruits harvested in Winter of 2017 was received as a generous gift from Ventura Coastal, LLC (California, USA). Enzymatic hydrolysate of orange peel was prepared as previously described, with modification. Peel solids diluted in water (500 g L^−1^) were hydrolyzed for 24 h at room temperature by addition of pectinase (Sigma 17389) and cellulase (Sigma C2730) added at 0.5 mg enzyme per grams of total peel solids^21^. For growth assays, hydrolysate was filtered through a fine mesh strainer and centrifuged before addition of 6.7 g L^−1^ Difco yeast nitrogen base w/o amino acids, 2 g L^−1^ synthetic defined amino acid mix, w/o yeast nitrogen base (US Biological) and sterilized by filtration. For *meso-*galactaric acid production, 2 g L^−1^ Difco yeast nitrogen base w/o amino acids, 2 g L^−1^ synthetic defined amino acid mix, w/o yeast nitrogen base (US Biological) and 100 g L^−1^ d-glucose, where indicated, was added. Sterile water was added to normalize sugar concentrations among hydrolysate batches. Growth of catabolic pathway strains in CPW hydrolysate media was performed in 50 mL cultures in 250 mL baffled flasks with 30 °C at 18 × *g* shaking in a New Brunswick™ Innova® 44 shaking incubator.

### Plasmids and cloning

A hierarchical golden gate cloning scheme was used for assembling coding sequence part plasmids, yeast protein expression cassettes and multigene plasmids^70^. All protein coding sequences were synthesized or PCR amplified to omit internal BsaI and BsmBI sites for use in golden gate cloning. The protein coding sequences for fungal pathway enzymes were codon optimized for *S. cerevisiae* and synthesized Integrated DNA technologies (Coralville, IA). The protein coding sequences for uronate dehydrogenase from *Pseudomonas syringae* pv. tomato str. DC3000 and GAT-1 homologues were PCR amplified from genomic DNA or cDNA of respective fungal hosts. Single gene yeast expression plasmids for fungal enzymes (pRJP010, pRJP912, pRJP913, pER046, pRJP138, pRJP157, pER047, pRJP042, pRJP914, and pZYM005) and transporters (pRJP869-pRJP871, pRJP918-pRJP923, and pRJP1312) were cloned by BsaI golden gate reaction with respective yeast promoter and terminator pairs (Supplementary Data 2) into a backbone containing ColE1 bacterial replication origin, Kanamycin resistance for bacterial selection and the respective yeast selection marker. Transporters from *Botrytis cinerea B05.10* and *Aspergillus nidulans* were PCR amplified from cDNA with overhangs homologous to a yeast expression cassette (pTDH3-tADH1) targeting the Leu2 locus. PCR products and homologous yeast expression fragments were co-transformed into yeast for assembly by homologous recombination and colonies were selected for on SC-Leu dropout plates and confirmed by colony PCR. Multigene yeast expression plasmids (pRJP707, pRJP734, and pRJP915-917) were assembled from single gene cassettes in BsmBI golden gate reactions with their respective bacterial and yeast origins of replication and selection markers (Supplementary Table 3). Primers used for *Neurospora* complementation strains cloning were list in Supplementary Data 3.

### Phylogenetic analysis

Fungal homologues of GAT-1, (NCU06026, quinate permease), from representative ascomycete and basidiomycete species were identified by BLAST and retrieved from the NCBI database. Protein sequences were aligned by Phylogeny.fr^71^ and a phylogenetic tree constructed using the program FigTree (http://tree.bio.ed.ac.uk/software/figtree/). Gal2p and Hxt2p from *S. cerevisiae* were used as out-groups.

### Sugar consumption and conversion assays and HPLC conditions

Yeast strains were grown to OD_600_ = 1.0 in YPD or selective media, harvested and washed twice in sterile water. Cells were resuspended in assay media at the stated OD in 3 mL cultures and incubated in 24-deep well culture blocks at 30 °C at 16 × *g* in an INFORS HT Multitron shaker with air-permeable seals. For *meso-*galactaric acid production assay in CPW hydrolysate media, strains were grown in YPD media overnight to saturation before washing twice in sterile water, pelleted and suspended in respective CPW hydrolysate media. Fermentations were conducted in 250 mL baffled flasks with 30 °C at 18 × *g* shaking in a New Brunswick™ Innova® 44 shaking incubator. Batch feeding of d-glucose was performed by adding either a super-saturated 1000 g L^−1^ d-glucose solution or sterile water. For determination of media sugar concentrations, culture aliquots were pelleted and the supernatant was transferred to GC/MS vials for sampling. Fermentation samples for *meso-*galactaric acid analysis were adjusted to pH 10 with NaOH to solubilize sugar acids before HPLC analysis.  Media samples were analyzed by refractive index on a Shimadzu LC20AD HPLC equipped with a Rezex RFQ-Fast Acid H^+^ (8%), LC Column (100 × 7.8 mm) run with 0.5 mL per min 0.01 N H_2_SO_4_ mobile phase at 65 °C. Sugar concentrations were determined by comparing HPLC traces to a standard curve.

### (^3^H)-Galacturonic acid transport kinetics in yeast

Transport assays as described in Benz et al., with modification^23^. Yeast strains were grown to an OD_600_ of 1.0 in YPD media, washed three times with ice cold assay buffer 30 mM MES-NaOH and resuspended to an OD of 80.0. To start transport reactions, cells were added to various (^3^H)-d-galUA concentrations and incubated at 30 °C. At respective time points, 100 μL of cell mixture was layered over 100 μL of silicone oil (Sigma 85419) and reactions were stopped by spinning cells through oil for 1 min at 17,000 × *g*. Each tube was frozen in a bath of ethanol and dry ice and tube-bottoms containing the cell pellets were clipped off into vials containing 5 mL of Ultima Gold scintillation fluid. Cell solutions were solubilized at room temperature overnight and counts per minute (CPM) determined in a Tri-Carb 2900TR scintillation counter. (^3^H)-d-galUA was purchased from ViTrax, Inc. (Placentia, CA, USA), and had a specific activity of 25 Ci per mmol and a purity of > 99%. Kinetic parameters were determined by measuring the linear rate of (^3^H)-d-galUA uptake over 5 min for d-galUA concentrations between 50 and 1000 μM. Vmax values were normalized by total protein content using a Bradford assay with bovine serum albumin as a standard.

## Electronic supplementary material

Supplementary Information

Description of Additional Supplementary Files

Supplementary Data 1

Supplementary Data 2

Supplementary Data 3

Reporting Summary

## Data Availability

A reporting summary for this Article is available as a Supplementary Information file. The data that support the findings of this study are available from the corresponding author upon reasonable request.
