# Supplementary material for: Engineering Saccharomyces cerevisiae for co-utilization of d-galacturonic acid and d-glucose from citrus peel waste
Source: Nat Commun. 2018 Nov 29;9:5059. doi: 10.1038/s41467-018-07589-w (PMC6265301; doi:10.1038/s41467-018-07589-w)
Supplement: Supplementary file 1 — Supplementary Information [file 41467_2018_7589_MOESM1_ESM.docx]

**Engineering *Saccharomyces cerevisiae* for co-utilization of D-galacturonic acid and D-glucose from citrus peel waste**

**Protzko *et al.***


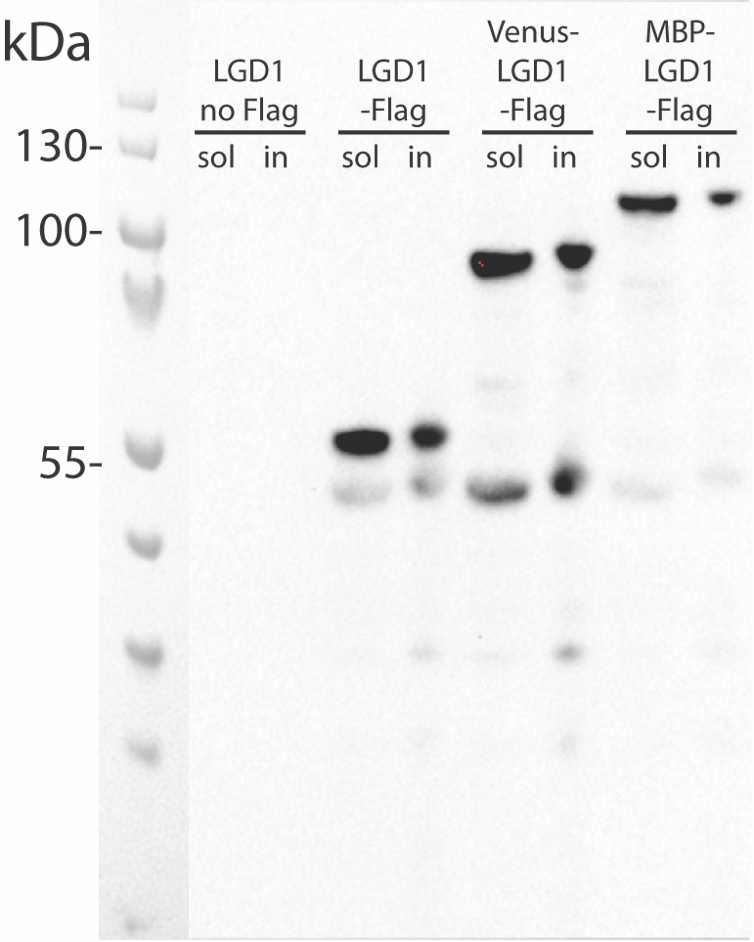


**Supplementary Figure 1.** N-terminal tagging of LGD1 does not qualitatively improve protein solubility. Anti-FLAG western blot of soluble (sol) and insoluble (in) fractions of yeast protein lysates C-terminal FLAG epitope tagged constructs of LGD1 alone (LGD1-FLAG, yRJP207), N-terminally fused LGD1 with Venus (Venus-LGD1-FLAG, yRJP210) and N-terminally fused LGD1 with maltose-binding protein (MBP-LGD1-FLAG, yRJP209). LGD1 without a FLAG fusion tag (yER022) shows no anti-FLAG signal.


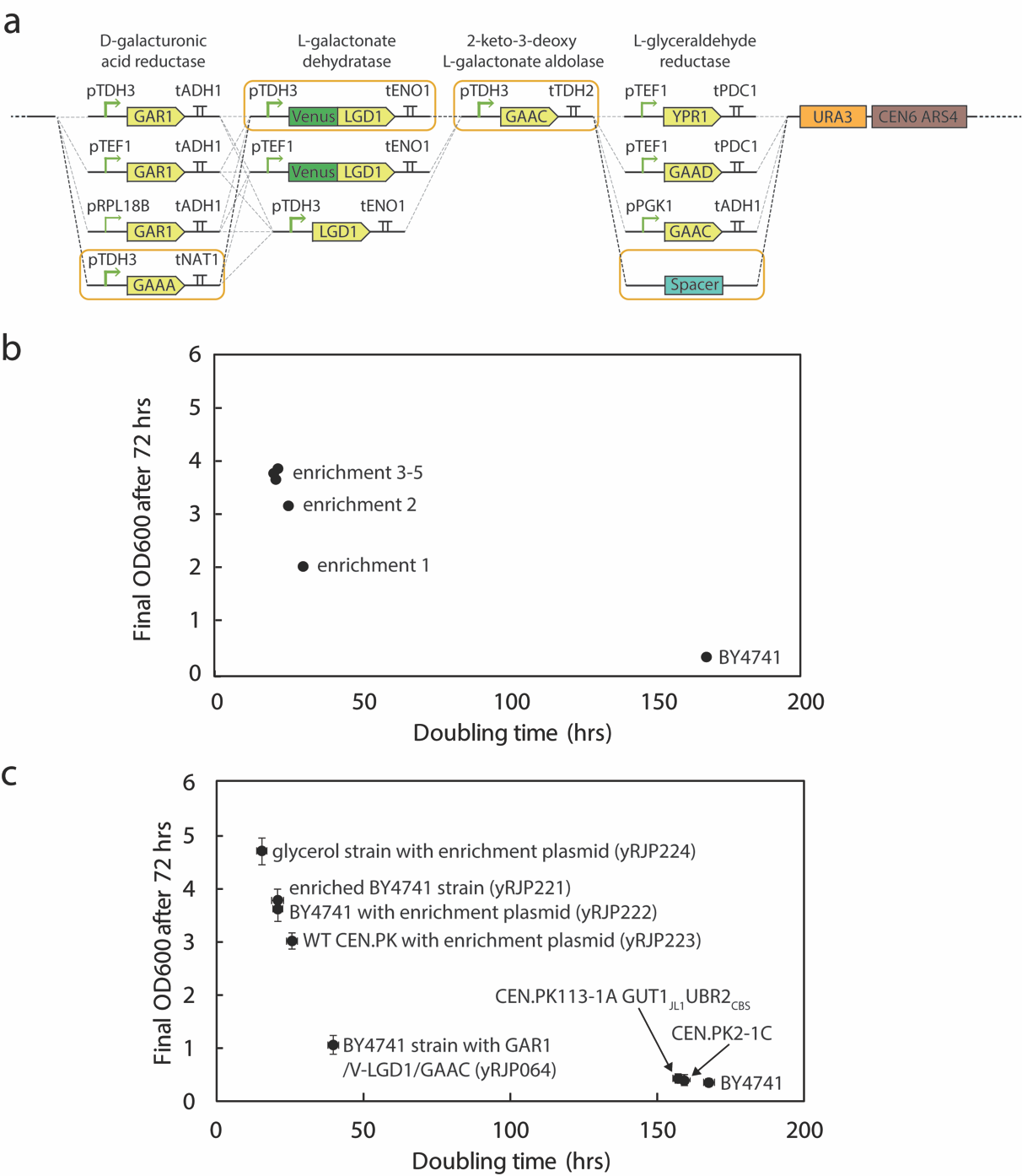


**Supplementary Figure 2.** Isolation of a *S. cerevisiae strain* capable of growth on D-galUA liquid media. (A) A combinatorial library sampling multiple expression levels of pathway enzymes, cofactor usage by D-galacturonic acid reductase, L-galactonate dehydratase with and without a fusion to Venus, and various orthologs or absence of L-glyceraldehyde reductase was assembled into a low-copy plasmid. (B) The combinatorial library was transformed into wild-type BY4741 and subjected to 5 rounds of enrichment in shake flasks by serial back dilution to OD 0.3 in fresh D-galUA media every 72 hours. After 3 rounds of enrichment, the culture doubling time and final density stabilized. Optical cell density and highest calculated doubling time were determined from 72 hour growth curves. (C) The enriched strain of the combinatorial library contained a multigene plasmid (pRJP1444) with GAAA, Venus-LGD1 and GAAC at the highest-strength promoter (*pTDH3*) levels (in orange boxes in SI Fig 2A). The enriched strain (yRJP221) has identical growth profiles in D-galUA media as a wild-type BY4741 strain transformed with the enriched plasmid (yRJP222) and exhibits higher final OD and doubling times than a BY4741 strain with GAR1, Venus-LGD1 and GAAC at *pTDH3* levels (yRJP064). Inclusion of the enriched plasmid in strain CEN.PK113-1A GUT1_JL1_UBR2_CBS_ engineered for glycerol utilization (yRJP224) shows higher final OD and doubling times than a wild-type CEN.PK strain containing the enrichment plasmid (yRJP223). Error bars represent standard deviation of biological triplicates.

.
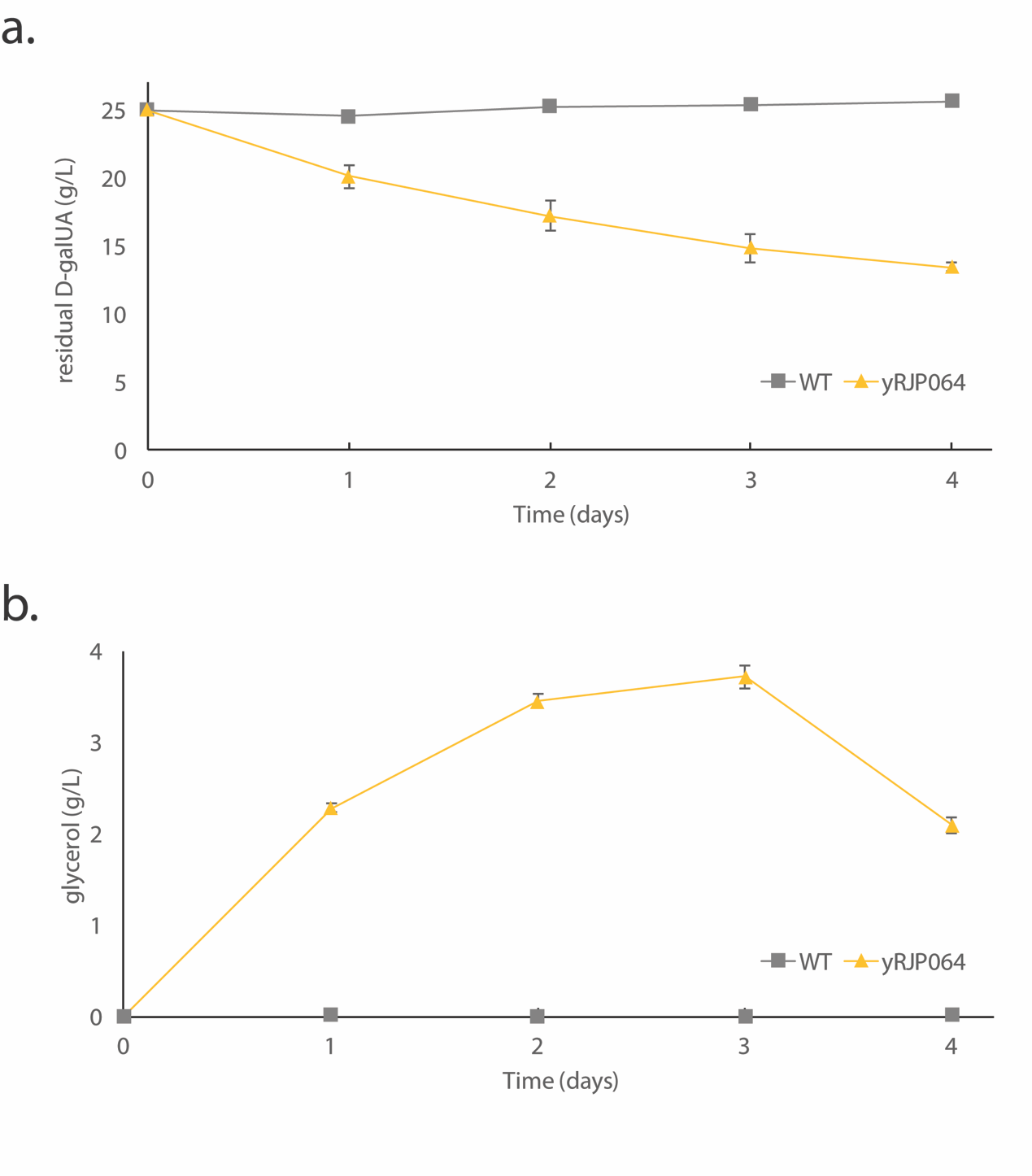


**Supplementary Figure 3.** BY4741 containing a heterologous D-galUA utilization pathway (yRJP064) accumulates glycerol in the media during incubation with D-galUA media. Cultures of yRJP064 (orange triangles) expressing high activity enzymes for the fungal D-galUA catabolism pathway and wild-type BY4741 (grey squares) were grown to OD_600_ = 10 on D-glucose and transferred to synthetic defined medium containing 25 g/L D-galUA as the sole carbon source. Residual D-galUA (A) and production of glycerol (B) in the media was monitored by HPLC over several days. Error bars represent standard deviation of biological triplicates.


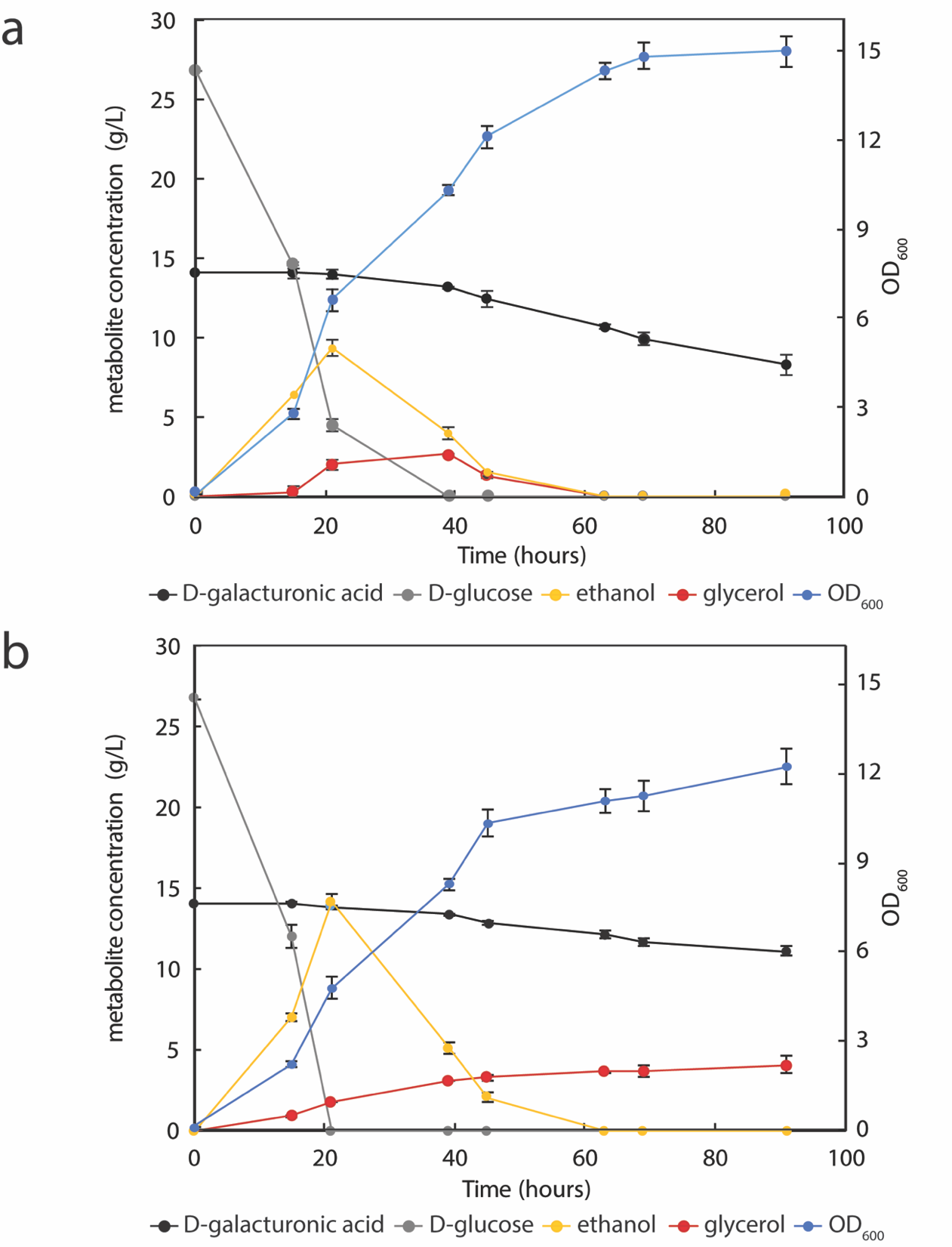


**Supplementary Figure 4.** Engineered glycerol utilization improves final cell density (blue), total D-galUA utilization (black) and eliminates accumulation of glycerol during growth on citrus peel waste hydrolysate compared to wild-type (red). (A) Aerobic growth curves and media metabolite concentrations for a CEN.PK strain engineered for glycerol utilization and expressing the optimized D-galUA utilization pathway, (yRJP224). (B) Aerobic growth curve media contents for a wild-type CEN.PK strain with the optimized D-galUA utilization pathway, yRJP223. Error bars represent standard deviation of biological triplicates.


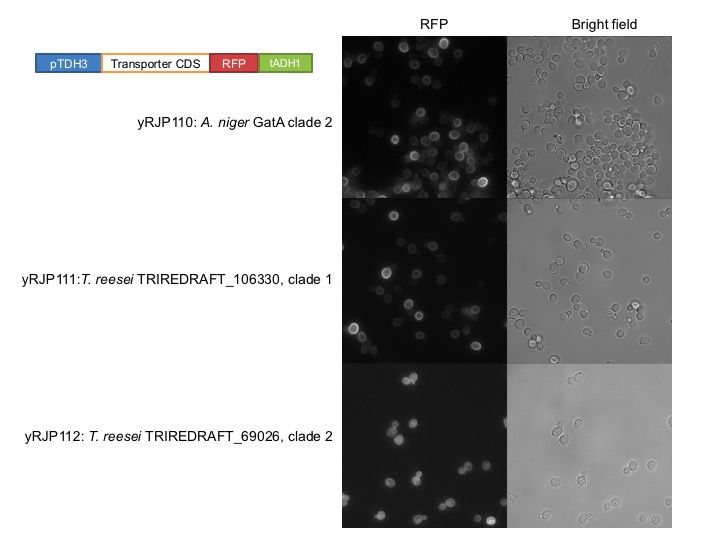


**Supplementary Figure 5.**  The C-terminally RFP-tagged GAT-1 homologs, *A. niger* GatA, *T. reesei* TRIREDRAFT_106330 and *T. reesei* TRIREDRAFT_69026, exhibiting transport activity of D-galacturonic acid at pH 5.5, also exhibit plasma membrane trafficking in yeast. Strains were grown to mid-log in synthetic complete dextrose media and washed with PBS before monitoring cells for RFP fluorescence and bright field microscopy at 100x magnification.


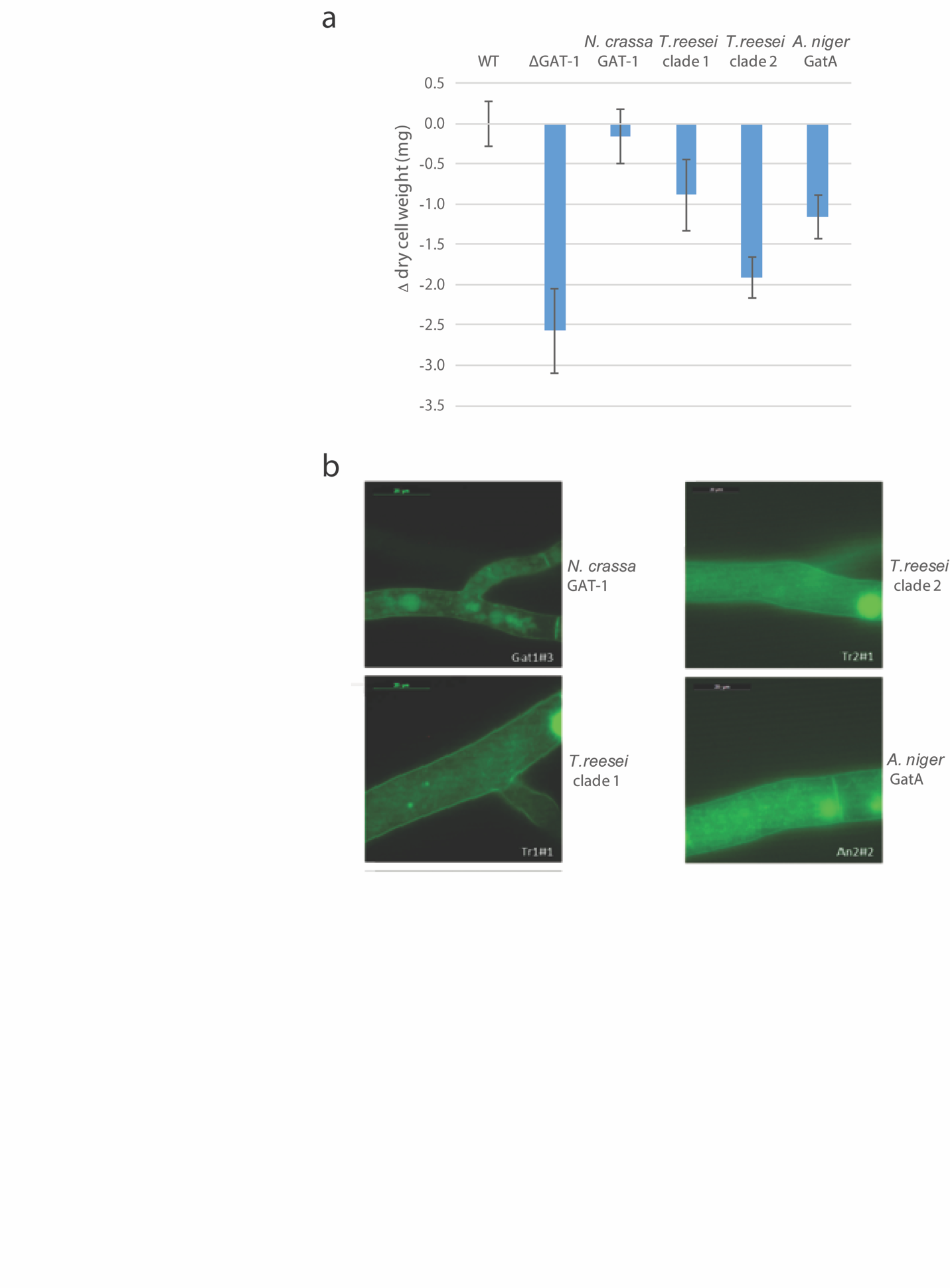


**Supplementary Figure 6.**  C-terminally GFP-tagged GAT-1 homologs expressed in the *N. crassa* Δ*gat-1* background partially complement the growth phenotype on pectin. (A) Strains were grown for four days on 1% citrus-peel pectin to measure biomass of several individual complementation lines in comparison to the parental line (Δ*GAT-1*) and wild-type. Difference in final dry cell weight (mg) compared to wild-type (WT) was plotted and error bars represent standard deviation of 5-9 biological replicates. (B) GFP fluorescence of representative transformants indicate correct targeting to the plasma membrane, as well as non-specific and vacuolar signals due to artificial overexpression by the GPD promoter.


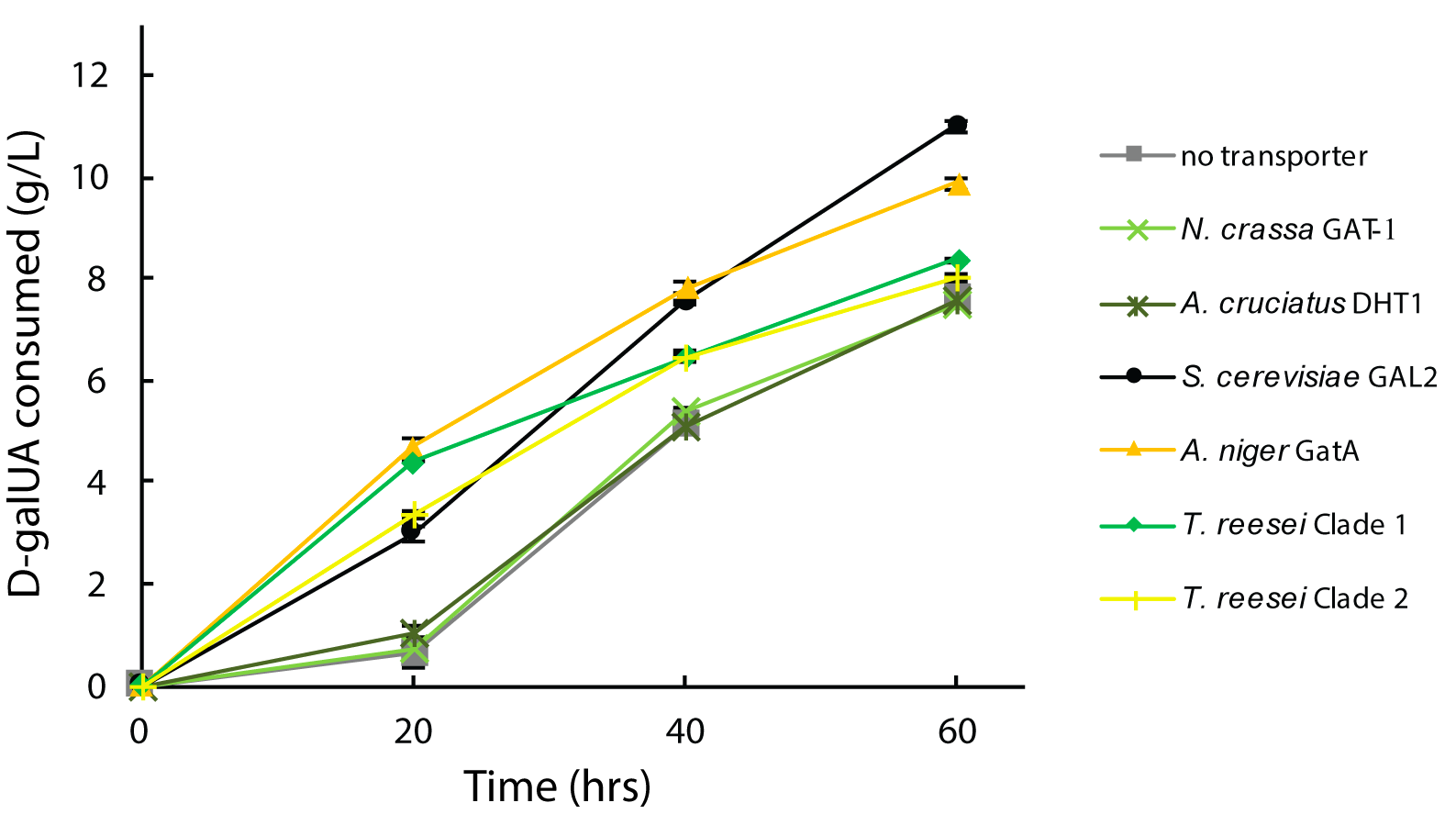


**Supplementary Figure 7.** Consumption of D-galUA at pH 3.5 is retained for all strains expressing different GAT-1 transporter homologs. Consumption of D-galUA (starting concentration of 20 g/L) in pH 3.5 buffered D-galUA media was monitored by HPLC in yRJP064 strains expressing various GAT-1 homologue transporters (OD_600_ = 10). Error bars represent standard deviation of biological triplicates.


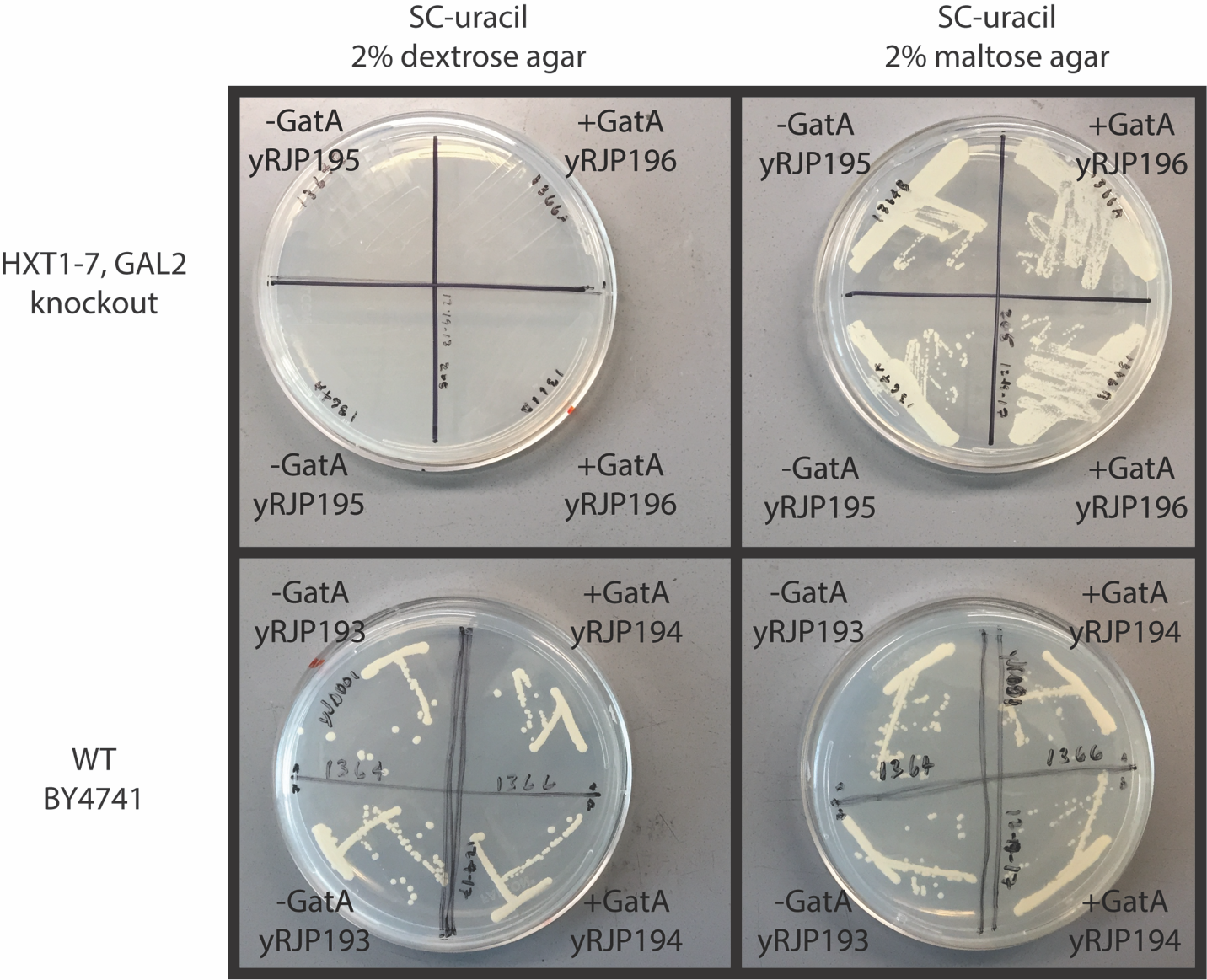


**Supplementary Figure 8.** GatA does not complement growth of hexose transporter knockout strain on D-glucose. Wild-type BY4741 expressing the D-galUA fungal catabolism pathway without (yRJP193) and with GatA (yRJP194) and KY73 (ΔHXT1-7, ΔGAL2) expressing the D-galUA fungal catabolism pathway without (yRJP195) and with GatA (yRJP196) grown on synthetic complete media without uracil with maltose or D-glucose as a carbon source. yRJP196 expressing GatA does not exhibit growth on D-glucose.


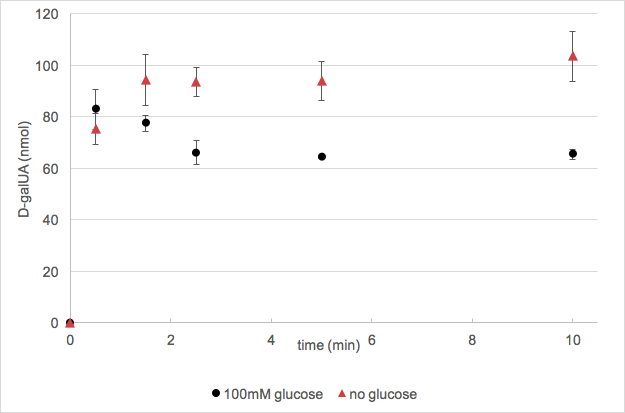


**Supplementary Figure 9.** Contrary to fermentation studies, rapid association of tritiated D-galUA with wild-type yeast is observed in both the presence and absence of D-glucose by biochemical assay. Wild-type BY4741 yeast cells were preincubated with buffer (no glucose, red triangles) or 100 mM D-glucose (black circles) before uptake of 50 mM tritiated D-galacturonic acid solution was monitored over several minutes at pH 3.5. Error bars represent standard deviation of technical triplicates.


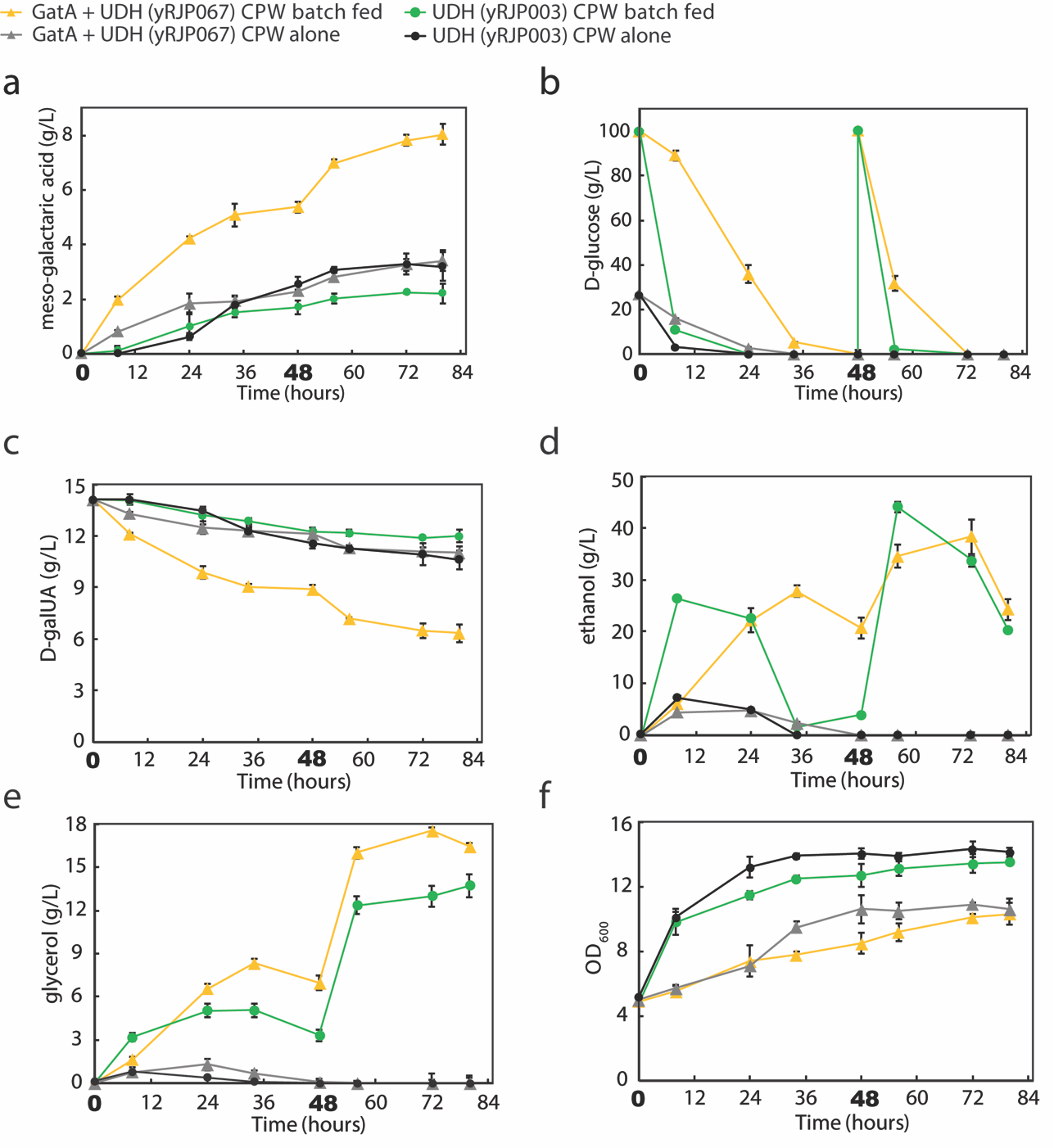


**Supplementary Figure 10.** Strains co-expressing UDH and GatA (yRJP067, grey and yellow triangles) produce more *meso-*galactaric acid (A) in CPW hydrolysate media in the presence of D-glucose (B) compared to strains expressing only UDH (yRJP003, black and green circles). Batch feeding of D-glucose at 0 and 48 hrs (bolded in x-axis) increases D-galUA consumption (C) when expressing GatA (yellow triangles), but inhibits consumption without GatA (green circles). (D) Ethanol accumulates as a coproduct in batch fed D-glucose cultures (orange triangles, green circles) compared to unsupplemented CPW hydrolysate cultures (grey triangles, black circles). (E) Higher glycerol accumulation is observed in respective culture conditions for the UDH and GatA co-expression strain (yellow and grey triangles) compared to UDH alone strain (black and green circles), indicating greater oxidation activity from UDH. (F) Strains expressing GatA (yellow and grey triangles) show lower optical densities than those only expressing UDH (black and green circles). Concentrations were normalized following glucose addition. Error bars represent standard deviation of biological triplicates.


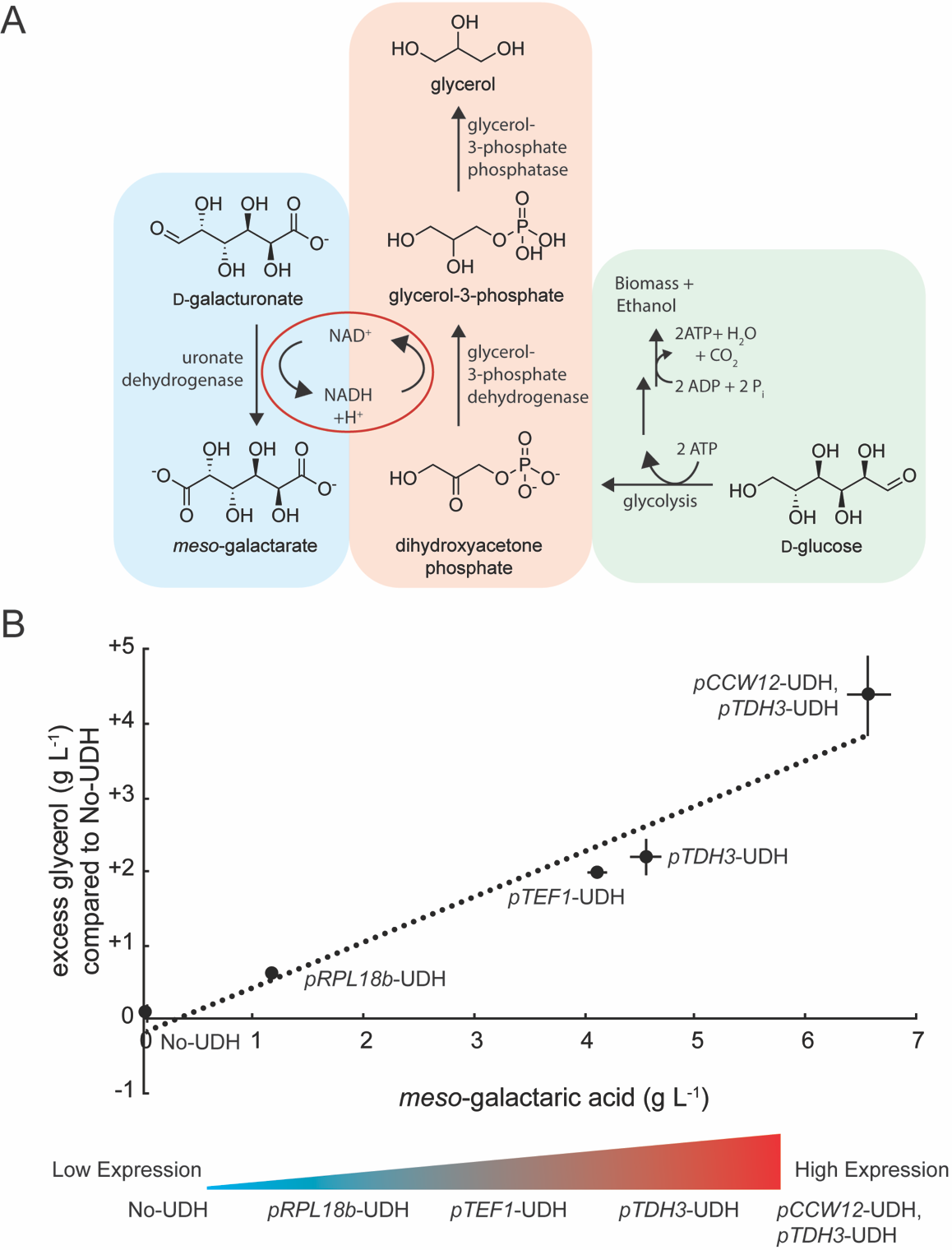


**Supplementary Figure 11.** Increasing uronate dehydrogenase (UDH) expression results in higher *meso-*galactarate and glycerol formation in yeast cultures co-fed D-glucose and D-galUA. (A) Redox couple between UDH oxidation and glycerol-3-phosphate dehydrogenase reduction activities (red circle) is formed when D-galacturonic acid and D-glucose is co-fed to cells. (B) Cultures of yeast expressing increasing levels^1^ of UDH from no enzyme (No*-*UDH, yRJP235) to highest enzyme (two copies of UDH at *pTDH3*-UDH, *pCCW12*-UDH, yRJP238) show increased glycerol and *meso-*galactaric acid formation when co-fed D-galacturonic acid and D-glucose. Error bars represent standard deviation of technical triplicates.

**Supplementary Table 1.** Description and design of strains for heterologous expression of single genes in yeast.*

| **Strain name** | **Strain background** | **Plasmid number** | **Enzyme Description** | **Organism** | **Gene Name** | **Expression design** | **Marker** | **Codon**  **opt** |
| --- | --- | --- | --- | --- | --- | --- | --- | --- |
| yER020 | BY4741 | pRJP010 | D-galacturonic acid reductase | *Aspergillus niger* | GAAA | pTDH3-GAAA-tADH1 | LEU2 | yes |
| yER021 | BY4741 | pER046 | L-galactonate dehydratase | *Neurospora crassa* | NCU07064 | pTDH3-NCU07064-tADH1 | LEU2 | yes |
| yER022 | BY4741 | pRJP138 | L-galactonate dehydratase | *Trichoderma reesei* | LGD1 | pTDH3-LGD1-tADH1 | LEU2 | yes |
| yER023 | BY4741 | pRJP157 | L-galactonate dehydratase | *Aspergillus niger* | GAAB | pTDH3-GAAB-tADH1 | LEU2 | yes |
| yER024 | BY4741 | pER047 | 2-keto-3-deoxy-L-galactonate aldolase | *Neurospora crassa* | NCU09532 | pTDH3-NCU09532-tADH1 | LEU2 | no |
| yER025 | BY4741 | pRJP042 | 2-keto-3-deoxy-L-galactonate aldolase | *Trichoderma reesei* | LGA1 | pTDH3-LGA1-tADH1 | LEU2 | yes |
| yER026 | BY4741 | pTMP122 | Marker Control Plasmid | n.a. | n.a. | n.a. | URA3 | *n.a.* |
| yRJP001 | BY4741 | pTMP130 | Marker Control Plasmid | n.a. | n.a. | n.a. | LEU2 | *n.a.* |
| yRJP003 | BY4741 | pZYM004 | uronate dehydrogenase | *Pseudomonas syringae* | UDH | pTDH3-UDH-tTDH1 | URA3 | no |
| yRJP058 | BY4741 | pRJP912 | D-galacturonic acid reductase | *Trichoderma reesei* | GAR1 | pCCW12-GAR1-tADH1 | URA3 | yes |
| yRJP059 | BY4741 | pRJP913 | L-galactonate dehydratase | *Trichoderma reesei* | Venus-LGD1 | pTDH3-Venus-LGD1-tENO1 | URA3 | yes |
| yRJP060 | BY4741 | pRJP914 | 2-keto-3-deoxy-L-galactonate aldolase | *Aspergillus niger* | GAAC | pTDH3-GAAC-tADH1 | URA3 | yes |
| yRJP207 | BY4741 | pRJP1415 | L-galactonate dehydratase | *Trichoderma reesei* | LGD1-FLAG | pTDH3-LGD1-FLAG-tADH1 | LEU2 | yes |
| yRJP208 | BY4741 | pRJP1416 | L-galactonate dehydratase | *Trichoderma reesei* | Venus-LGD1-FLAG | pTDH3-Venus-LGD1-FLAG-tADH1 | LEU2 | yes |
| yRJP209 | BY4741 | pRJP1417 | L-galactonate dehydratase | *Trichoderma reesei* | MBP-LGD1-FLAG | pTDH3-MBP-LGD1-FLAG-tADH1 | LEU2 | yes |
| yRJP226 | BY4741 | pRJP1418 | L-galactonate dehydratase | *Trichoderma reesei* | MBP-LGD1 | pTDH3-MBP-LGD1-tADH1 | LEU2 | yes |

*Codon optimization (codon opt) for *S. cerevisiae* are listed for each CDS (“yes” indicates codon optimization and “no” indicates native CDS). Plasmids contain ColE1 for bacterial replication, kanamycin resistance marker for bacterial selection and CEN6/ARS4 elements for yeast replication.

**Supplementary Table 2.** Specific activities of single gene expression cassettes as measured in cell lysate.*

| **Strain number** | **Enzyme description** | **Gene Name** | **Specific activity (µmol min^-1^ mg^-1^ protein)** | **K_m_ (mM)** |
| --- | --- | --- | --- | --- |
| yER020 | *A. niger* D-galacturonic acid reductase | GAAA | 0.0355 (NADPH) |  |
| yER020 | *A. niger* D-galacturonic acid reductase | GAAA | 0.0245 (NADH) |  |
| yRJP058 | *T. reesei* D-galacturonic acid reductase | GAR1 | 0.1318 |  |
| yRJP059 | *T. reesei* Venus*-*L-galactonate dehydratase | Venus-LGD1 | 0.0665 | 5.1 ± 0.4 |
| yRJP226 | *T. reesei* Maltose Binding Protein*-*L-galactonate dehydratase | MBP-LGD1 | 0.0059 |  |
| yER021 | *N. crassa* L-galactonate dehydratase | NCU07064 | none detected |  |
| yER009 | *T. reesei* L-galactonate dehydratase | LGD1 | 0.0011 | 4.9 ± 0.2 |
| yER023 | *A. niger* L-galactonate dehydratase | GAAB | none detected |  |
| yER024 | *N. crassa* 2-keto-3-deoxy-L-galactonate aldolase | NCU09532 | 0.0189 |  |
| yER025 | *T. reesei* 2-keto-3-deoxy-L-galactonate aldolase | LGA1 | 0.0319 |  |
| yRJP060 | *A. niger* 2-keto-3-deoxy-L-galactonate aldolase | GAAC | 0.0711 |  |

*The average of biological duplicates is shown. Km values were determined for untagged and N-terminally Venus tagged *T. reesei* L-galactonate dehydratase (LGD1) strains.

**Supplementary Table 3.** Description and design of strains containing multigene expression cassettes for the fungal catabolism pathway.*

| **Strain name** | **Strain background** | **Plasmid number** | **Expression Design** | **Marker** |
| --- | --- | --- | --- | --- |
| yRJP061 | BY4741 | pRJP915 | pCCW12-GAR1-tADH1 - pTDH3-Venus-LGD1-tENO1 - spacer | URA3; CEN/ARS4 yeast replication |
| yRJP062 | BY4741 | pRJP916 | pCCW12-GAR1-tADH1 - pTDH3-GAAC-tTDH2 - spacer | URA3; CEN/ARS4 yeast replication |
| yRJP063 | BY4741 | pRJP917 | Spacer - pTDH3-Venus-LGD13b-tENO1 - pTDH3-GAAC-tTDH2 | URA3; CEN/ARS4 yeast replication |
| yRJP064 | BY4741 | pRJP707 | pCCW12-GAR1-tADH1 - pTDH3-Venus-LGD1-tENO1 - pTDH3-GAAC-tTDH2 | URA3; CEN/ARS4 yeast replication |
| yRJP193 | BY4741 | pRJP1364 | pCCW12-GAR1-tADH1 - pTDH3-Venus-LGD1-tENO1 - pTDH3-GAAC-tTDH2 - spacer | URA3; CEN/ARS4 yeast replication |
| yRJP194 | BY4741 | pRJP1366 | pCCW12-GAR1-tADH1 - pTDH3-Venus-LGD1-tENO1 - pTDH3-GAAC-tTDH2 - pTEF-GatA-tPGK1 | URA3; CEN/ARS4 yeast replication |
| yRJP195 | KY73 | pRJP1364 | pCCW12-GAR1-tADH1 - pTDH3-Venus-LGD1-tENO1 - pTDH3-GAAC-tTDH2 - spacer | URA3; CEN/ARS4 yeast replication |
| yRJP196 | KY73 | pRJP1366 | pCCW12-GAR1-tADH1 - pTDH3-Venus-LGD1-tENO1 - pTDH3-GAAC-tTDH2 - pTEF-GatA-tPGK1 | URA3; CEN/ARS4 yeast replication |
| yRJP221 | BY4741 - enriched from D-galUA combinatorial library | pRJP1444 | pTDH3-GAAA-tNAT1 - pTDH3-Venus-LGD1-tENO1 - pTDH3-GAAC-tTDH2 | URA3; CEN/ARS4 yeast replication |
| yRJP222 | BY4741 | pRJP1444 | pTDH3-GAAA-tNAT1 - pTDH3-Venus-LGD1-tENO1 - pTDH3-GAAC-tTDH2 | URA3; CEN/ARS4 yeast replication |
| yRJP223 | CEN.PK2-1C | pRJP1444 | pTDH3-GAAA-tNAT1 - pTDH3-Venus-LGD1-tENO1 - pTDH3-GAAC-tTDH2 | URA3; CEN/ARS4 yeast replication |
| yRJP224 | CEN.PK113-1A GUT1_JL1_UBR2_CBS_ | pRJP1444 | pTDH3-GAAA-tNAT1 - pTDH3-Venus-LGD1-tENO1 - pTDH3-GAAC-tTDH2 | URA3; CEN/ARS4 yeast replication |

*Plasmids contain ColE1 and kanamycin resistance elements for bacterial replication and selection.

**Supplementary Table 4.** Plasmid designs for combinatorial expression and enzyme library sampling multiple expression levels of pathway enzymes, cofactor usage by D-galacturonic acid reductase, L-galactonate dehydratase with and without a fusion to Venus, and various orthologs or absence of L-glyceraldehyde reductase.*

| **Assembly position** | **Plasmid name** | **Enzyme description** | **Gene name** | **Expression cassette design** |
| --- | --- | --- | --- | --- |
| Position 1 (conLS-conR1) | pRJP545 | *A. niger* D-galacturonic acid reductase | GAAA | pTDH3-GAAA-tNAT1 |
|  | pRJP562 | *T. reesei* D-galacturonic acid reductase | GAR1 | pCCW12-GAR1-tADH1 |
|  | pRJP563 | *T. reesei* D-galacturonic acid reductase | GAR1 | pTEF1-GAR1-tADH1 |
|  | pRJP564 | *T. reesei* D-galacturonic acid reductase | GAR1 | pRPL18B-GAR1-tADH1 |
| Position 2 (conL1-conR2) | pRJP565 | *T. reesei Venus-*L-galactonate dehydratase | Venus-LGD1 | pTDH3-Venus-LGD1-tENO1 |
|  | pRJP566 | *T. reesei Venus-*L-galactonate dehydratase | Venus-LGD1 | pTEF2-Venus-LGD1-tENO1 |
|  | pRJP708 | *T. reesei* L-galactonate dehydratase | LGD1 | pTDH3-LGD1-tENO1 |
| Position 3 (conL2-conR3) | pRJP567 | *A. niger* 2-keto-3-deoxy-L-galactonate aldolase | GAAC | pTDH3-GAAC-tTDH2 |
| Position 4 (conL3-conRE) | pTMP172 | *n.a.* | *n.a.* | spacer |
| Position 4 (conL3-conR4) | pRJP569 | *S. cerevisiae* aldo-keto reductase | YPR1 | pTEF1-YPR1-tPDC1 |
|  | pRJP571 | *A. niger* L-glyceraldehyde reductase | GAAD | pTEF1-GAAD-tPDC1 |
|  | pRJP813 | *A. niger* 2-keto-3-deoxy-L-galactonate aldolase | GAAC | pPGK1-GAAC-tADH1 |
| Position 4 (conL4-conRE) | pTMP173 | *n.a.* | *n.a.* | spacer |

*All plasmids contain an ampicillin resistance and ColE1 element for selection and replication in bacteria. Assembly positions are defined by flanking connector (con) regions containing unique BsmBI overhangs that flank each cassette and allow for assembly with complementary connector regions (e.g. ConS-Con1-Con2-Con3-Con4-ConE) into a multigene plasmid^1^.

**Supplementary Table 5.** Description and design of strains containing transporter expression cassettes.*

| **Strain name** | **Strain background** | **Plasmid number** | **Organism** | **Gene Name** | **Expression design** | **Clade** | **Codon opt** |
| --- | --- | --- | --- | --- | --- | --- | --- |
| yRJP048 | yRJP064 | pRJP870 | *Asteromyces cruciatus* | DHT1 | pTDH3-DHT1-tADH1 | 1 | yes |
| yRJP054 | yRJP064 | pRJP871 | *Saccharomyces cerevisiae* | Gal2p | pTDH3-Gal2-tADH1 | n.a. | n.a. |
| yRJP088 | yRJP064 | n.a. | *Botrytis cinerea B05.10* | BC1G_10717 | pTDH3-BC1G_10717-mRuby-tADH1 | 1 | no |
| yRJP089 | yRJP064 | n.a. | *Botrytis cinerea B05.10* | BC1G_12561 | pTDH3-BC1G_12561-mRuby-tADH1 | 2 | no |
| yRJP090 | yRJP064 | n.a. | *Botrytis cinerea B05.10* | BC1G_00956 | pTDH3-BC1G_00956-mRuby-tADH1 | 3 | no |
| yRJP091 | yRJP064 | n.a. | *Aspergillus nidulans* | AN5734 | pTDH3-AN5734-mRuby-tADH1 | 1 | no |
| yRJP092 | yRJP064 | n.a. | *Aspergillus nidulans* | AN7667 | pTDH3-AN7667-mRuby-tADH1 | 2 | no |
| yRJP093 | yRJP064 | pRJP918 | *Aspergillus niger* | An14g04280, GatA | pTDH3-GatA-tADH1 | 2 | no |
| yRJP107 | yRJP064 | pRJP869 | *Neurospora crassa* | GAT-1 | pTDH3-GAT-1-ADH1 | 1 | yes |
| yRJP108 | yRJP064 | pRJP919 | *Trichoderma reesei* | TRIREDRAFT_106330 | pTDH3-TRIREDRAFT_106330-tADH1 | 1 | no |
| yRJP109 | yRJP064 | pRJP920 | *Trichoderma reesei* | TRIREDRAFT_69026 | pTDH3-TRIREDRAFT_69026-tADH1 | 2 | no |
| yRJP110 | yRJP064 | pRJP921 | *Aspergillus niger* | An14g04280, GatA | pTDH3-GatA-mRuby-tADH1 | 2 | no |
| yRJP111 | yRJP064 | pRJP922 | *Trichoderma reesei* | TRIREDRAFT_106330 | pTDH3-TRIREDRAFT_106330-mRuby-tADH1 | 1 | no |
| yRJP112 | yRJP064 | pRJP923 | *Trichoderma reesei* | TRIREDRAFT_69026 | pTDH3-TRIREDRAFT_69026-mRuby-tADH1 | 2 | no |
| yRJP113 | yRJP064 | pRJP1312 | *Rhodosporidium toruloidies* | RHTO_01744 | pTDH3-RHTO_01744-mRuby-tADH1 | 4 | yes |
| yRJP116 | yRJP064 | pTMP137 | n.a. | n.a. | Leu2 marker only plasmid control | n.a. | n.a. |

*GAT-1 homologue clade and codon optimization (codon opt) for *S. cerevisiae* are listed for each CDS (“yes” indicates codon optimization and “no” indicates native CDS). All plasmids contain ColE1 and kanamycin resistance elements for bacterial replication and selection. All plasmids are designed for integration into yeast genome at the LEU2 locus. Transporter expression DNA for yRJP088-092 was constructed by PCR and homologous recombination in yeast (see SI Materials and Methods section).

**Supplementary Table 6.** Description and design of yeast strains used for *meso-*galactaric acid production experiments.*

| **Strain name** | **Strain background** | **Strain description** |
| --- | --- | --- |
| yRJP003 | BY4741 | plasmid pZYM004 (pTDH3-uronate dehydrogenase-tTDH1) integrated into URA3 locus; plasmid pTMP137 (Leu2 marker alone) integrated into the LEU2 locus |
| yRJP067 | BY4741 | plasmid pZYM004 (pTDH3-uronate dehydrogenase-tTDH1) integrated into URA3 locus; plasmid yRJP918 (pTDH3-*A.nigers* GatA transporter-tADH1) integrated into the LEU2 locus |
| yRJP235 | BY4741 | plasmid pTMP138 (URA3 alone) integrated into URA3 locus; plasmid yRJP918 (pTDH3-*A.nigers* GatA transporter-tADH1) integrated into the LEU2 locus |
| yRJP236 | BY4741 | plasmid pRJP1245 (pRPL18B-uronate dehydrogenase-tTDH1) integrated into URA3 locus; plasmid yRJP918 (pTDH3-*A.nigers* GatA transporter-tADH1) integrated into the LEU2 locus |
| yRJP237 | BY4741 | plasmid pRJP1244 (pTEF1-uronate dehydrogenase-tTDH1) integrated into URA3 locus; plasmid yRJP918 (pTDH3-*A.nigers* GatA transporter-tADH1) integrated into the LEU2 locus |
| yRJP238 | BY4741 | plasmid pZYM005 (pTDH3-uronate dehydrogenase-tTDH1, pCCW12-uronate dehydrogenase-tENO1) integrated into URA3 locus; plasmid yRJP918 (pTDH3-*A.nigers* GatA transporter-tADH1) integrated into the LEU2 locus |

*Plasmids contain kanamycin resistance and ColE1 elements for bacterial resistance and replication.

**Supplementary Table 7.** Measurement of intracellular NAD^+^/NADH ratios in yeast strains containing no UDH (yRJP235) or high expression of UDH (yRJP067) after 4 hour incubation in synthetic media containing 100 g L^-1^ D-glucose alone, 20 g L^-1^ D-galUA alone, or 100 g L^-1^ D-glucose with 20 g L^-1^ D-galUA.*

| **Strain Name** | **Strain Description** | **Carbon Source in Media** | **NAD^+^/NADH Ratio** |
| --- | --- | --- | --- |
| yRJP235 | no UDH,  *pTDH3-*GatA | D-glucose | 1.42 ± 0.12 |
|  |  | D-galUA | 1.49 ± 0.07 |
|  |  | D-glucose + D-galUA | 1.53 ± 0.14 |
| yRJP067 | *pTDH3*-UDH,  *pTDH3-*GatA | D-glucose | 1.40 ± 0.06 |
|  |  | D-galUA | 0.89 ± 0.09 |
|  |  | D-glucose + D-galUA | 1.51 ± 0.13 |

*****High UDH activity cultures (yRJP067) show depletion of NAD^+^ in D-galUA alone media and higher NAD^+^/NADH levels with D-glucose supplementation. Average NAD^+^/NADH ratio and standard deviation represent technical triplicates.

**Supplementary Table 8.** Yeast strains expressing GatA and increasing expression levels of uronate dehydrogenase (UDH) were incubated (OD_600_= 20.0) with synthetic complete media containing 100 g L^-1^ D-glucose and 20 g L^-1^ D-galUA for 24 hours and the concentration of metabolites (g L^-1^) in media were assayed by HPLC.*

| **UDH Expression Level  (increasing promoter strength)** | **Strain**  **Name** | ***meso*-galactaric acid** | **stdev** | **D-galacturonic acid** | **stdev** | **glycerol** | **stdev** | **acetate** | **stdev** | **ethanol** | **stdev** |
| --- | --- | --- | --- | --- | --- | --- | --- | --- | --- | --- | --- |
| No UDH | yRJP235 | 0.00 | 0.000 | 19.56 | 0.01 | 10.9 | 0.1 | 3.83 | 0.04 | 35.0 | 0.9 |
| pRPL18b-UDH | yRJP236 | 1.15 | 0.01 | 18.93 | 0.01 | 11.4 | 0.1 | 3.69 | 0.02 | 35.02 | 0.03 |
| pTEF-UDH | yRJP237 | 4.10 | 0.09 | 16.44 | 0.06 | 12.79 | 0.04 | 4.46 | 0.04 | 34.2 | 0.7 |
| pTDH3-UDH | yRJP067 | 4.5 | 0.1 | 15.48 | 0.02 | 13.01 | 0.02 | 4.44 | 0.02 | 34.52 | 0.08 |
| pTDH3-UDH, pCCW12-UDH | yRJP238 | 6.5 | 0.2 | 13.47 | 0.03 | 15.20 | 0.05 | 3.01 | 0.01 | 33.2 | 0.3 |

*****In all cases, the D-glucose was fully consumed. A positive correlation between increasing *meso-*galactaric acid and glycerol concentrations are observed with increasing UDH expression levels. The average concentration of metabolites (g L^-1^) and standard deviation are shown representing biological triplicates.

**Supplementary Methods**

**Yeast lysate and protein sample preparations**

Yeast lysates for enzymatic assays and western blots were prepared by growing strains to OD_600_ of 1.0 in synthetic complete glucose media with the appropriate amino acid dropout, harvested by centrifugation and washed twice with cold assay buffer lacking substrate. Cells were concentrated to one hundredth the volume and subjected to bead beating in a Mini-Beadbeater-96 (Biospec) with zirconia/silica beads (0.5 mm diameter; Biospec, Bartlesville, OK, USA). Lysed cell mixture was separated from beads, centrifuged at 12,000x g for 10 min and the supernatant containing soluble protein used for enzymatic assays and soluble protein for western blotting. Insoluble protein fraction was prepared by suspending the pelleted protein mixture in buffer containing 1% SDS, boiling for 10 minutes, centrifugation at 12,000 xg and collecting the supernatant. Total protein was measured by Bradford assay with bovine serum albumin as a standard.

**Western blot**

Soluble and insoluble proteins samples prepared from strains yER022, yRJP207-209 in PAGE buffer were loaded (10ug/lane) onto a NuPAGE Novex 4–12% Bis-Tris gel (Life Technologies) and run for 2.5 h at 80 V. Proteins were transferred onto PVDF transfer membrane in NuPAGE transfer buffer (Life Technologies) and blocked overnight in TBST (0.05% Tween20) containing 5% milk. The membrane was washed 5x with TBST for 5 min and incubated for 1 h with HRP conjugated anti-Flag (Sigma no. A8592) monoclonal antibodies at a dilution of 1:5,000. After six 5-min washes in TBST, the HRP antibody was detected by chemiluminescence using a ChemiDoc XRS imager (Bio-Rad).

**Enzymatic assays**

Colorimetric absorbance detection assays were performed in Corning flat clear bottom 96 well plates on a Tecan Safire 2 plate reader. A continuous assay of D-galacturonic acid reductase activity of cell lysates was measured in a reaction mixture containing 100 mM phosphate buffer (pH 7.2), 100 mM NaCl, 10mM D-galacturonic acid and 1 mM NADPH. Initial velocity corresponding to NADPH oxidation was measured by loss of absorbance at 340 nm. End point assays for L-galactonate dehydratase activity in activity of cell lysates were measured in a reaction mixture containing 50 mM HEPES (pH 7.4), 100 mM NaCl, 5 mM MgCl_2_, and 10 mM L-galactonate. Detection of product was assayed at 540 nm using 3,5-dinitrosalicylic acid reagent (DNS) as described previously^2^. Reverse reaction of 2-keto-3-deoxy-L-galactonate aldolase activity in cell lysates were measured in a reaction mixture containing 50 mM HEPES (pH 7.4), 100 mM NaCl, 5 mM MgCl_2_, 10 mM pyruvate and 10 mM L-galactonate. Detection of product was assayed at 549 nm using a thiobarbaturic acid (TBA) assay as described previously^2^.

**Sugar consumption and conversion assays and HPLC conditions**

Yeast strains were grown to OD_600_ = 1.0 in YPD or selective media, harvested and washed twice in sterile water. Cells were resuspended in assay media at the stated OD in 3 mL cultures and incubated in 24-deep well culture blocks at 30 °C at 750 rpm in an INFORS HT Multitron shaker with air-permeable seals. For determination of media sugar concentrations, culture aliquots were pelleted and the supernatant was transferred to GC/MS vials for sampling. Media samples were analyzed by refractive index on a Shimadzu LC20AD HPLC equipped with a Rezex RFQ-Fast Acid H^+^ (8%), LC Column (100 x 7.8 mm) run with 0.5 mL/min 0.01 N H_2_SO_4_ mobile phase at 65°C. Sugar concentrations were determined by comparing HPLC traces to a standard curve.

**Construction of combinatorial promoter and enzyme library**

Single gene cassettes for library plasmids were cloned as described in the main text materials and methods. Each plasmid concentration was normalized by molar values before mixing in a one-pot BsmbI golden gate reaction. The library reaction was purified using a DNA Clean and Concentrator Kit (Zymo Research D4013) and eluted in water. The library DNA was transformed into TransforMax™ EPI300™ Electrocompetent *E. coli* (Lucigen EC300110) and plated on LB-kanamycin plates. These colonies were scraped from the plate and library DNA was isolated using a Zyppy Plasmid preparation kit (D4036). Library plasmid DNA (5 µg) was used to transform 5 mL of YPD grown OD_600_= 1.0 BY4741 yeast culture using the lithium acetate method. Transformed cells were plated onto synthetic complete dextrose media without uracil and incubated at 30 ^°^C for two days, yielding a 1x10^5^ transformant library at 1000x library coverage.

**Library culture enrichment conditions and strain growth conditions**

The combinatorial library was scraped from the plate with sterile water and diluted into SC-uracil 2% D-galUA (50 mL) at pH 3.5 to OD_600_ = 0.3. This culture was incubated at 250 RPM at 30 °C in 250mL glass baffled flasks in an INNOVA 44R Incubator Shaker (New Brunswick) until the culture reached OD_600_ = 1.0. The culture was then back diluted to OD_600_ = 0.3 every three days in the same media and incubation conditions. Culture density (OD_600_) was measured every 8-16 hours and doubling times were calculated using the least square fitting exponential method^3^. Final culture density (OD_600_) was measured 72 hours after the previous back dilution. Isolation of single colonies was performed by plating serial dilutions of the enrichment culture on SC-uracil glucose plates. For measurement of individual strain growths, strains were picked into 5 mL SC-uracil glucose media overnight at 30°C with 250 RPM shaking and back diluted to OD_600_ = 0.25 in SC-2% D-galUA media (50 mL) at pH 3.5 in 250 mL baffled flasks. Strain doubling and strain densities were calculated using the same methods as the culture enrichments.

**Cloning of *Neurospora* complementation strains**

*Neurospora crassa* WT (FGSC 2489), *Trichoderma reesei* QM6a (kind gift of the lab of Louise Glass, University of California at Berkeley) and *Aspergillus niger* WT (kind gift of the lab of John Gladden, JBEI) pre-grown on 2% glucose were switched for 16 hours to 0.5% citrus peel pectin (Sigma) before harvesting of the biomass. RNA was isolated from the harvested biomass by a Trizol protocol as described before^4^. From this, cDNA was prepared using the ProtoScript M-MuLV Taq RT-PCR kit (New England Biolabs). cDNA was used to amplify *gat-1* (NCU00988) and its respective homologs ANI_1_1412124 (An14g04280; *gatA*; *A. niger* clade 2), TRIREDRAFT_106330 (*T. reesei* clade 1) and TRIREDRAFT_69026 (*T. reesei* clade 2). See Supplementary Data 3 for primer information.

***Neurospora* complementation assays**

For complementation trials, between 5 and 10 individual, cyclosporine-resistant transformants were grown in biological triplicates next to the *N. crassa* WT and the parental Δ*gat-1* control in 3 mL 1% citrus peel pectin in 1x Vogel’s salts in 24-well deep-well plates at 200 rpm and 25 °C in the light. Inoculation was performed with about 9 day-old conidia at 1x10^6^ conidia/ml. After 4 days, biomass was harvested, washed and dried overnight at 105°C for dry weight determinations.

**Fluorescence Microscopy**

Yeast strains expressing GAT-1 homologue transporters C-terminally tagged with mRuby and transformants from the *Neurospora* Δgat-1 complementation experiments C-terminally tagged with GFP were furthermore analyzed by fluorescent microscopy using a Zeiss Axio Observer D1 microscope with a Zeiss Plan-Apochromat 100x oil immersion objective. Images were captured using a Hamamatsu Orca-flash 4.0 camera using 30 ms exposure for phase images and a X-Cite Series 120 lamp (Excelitas) and filter set 45 (Zeiss) using TurboYFP and mKate excitation/emission settings with a 400 ms exposure for GFP and mRuby fluorescence images, respectively.

**Measurement of intracellular ATP and NAD^+^/NADH Ratio**

Triplicate yeast (yRJP067) cultures were grown in synthetic complete glucose media lacking leucine and uracil until mid-log at 200 RPM at 30 °C in baffled flasks. Cells were pelleted, washed twice in sterile water and resuspended in either synthetic complete media with 100 g L^-1^ D-glucose alone, 20 g L^-1^ D-galUA alone or 20 g L^-1^ D-galUA and 100 g L^-1^ D-glucose and incubated for 4 hours at 30 °C. Measurement of yeast intracellular ATP was conducted as previously described^5^. In summary, cells were washed twice in cold PBS and a 100 µL suspension containing ~10^6^ cells was added to BactTiter-Glo^TM^ Microbial Cell Viability Assay solution according to the manufacturer’s protocol (Promega). Luminescence, which was proportional to intracellular ATP present, was recorded using TECAN Spark micro-plate reader. Measurement of yeast intracellular NAD^+^/NADH ratio was conducted as previously described^6^. In summary, 5 mL of the incubated cell culture was quenched in 20 mL 3:2 methanol/water in a dry ice acetonitrile bath. Cells were pelleted and washed twice with cold PBS before resuspension in NAD^+^ or NADH assay buffer and cell lysis by mechanical lysis (bead beating). NAD^+^/NADH ratios in lysates were measured using an Enzychrom NAD^+^/NADH assay kit (Bioassay Systems, Hayward, CA) according to the manufacturer’s instructions.

**Supplementary References**

1. Lee, M.E., Deloache, W.C., Cervantes, B., Dueber, J.E. A highly characterized yeast toolkit for modular, multipart assembly. *ACS Synth. Biol.* **4**, 975–986 (2015).
2. Hilditch, S. Identification of the fungal catabolic D-galacturonate pathway. University of Helinski, (2010).
3. Weisstein, Eric W. Least Squares Fitting--Exponential. *MathWorld*--A Wolfram Web Resource. http://mathworld.wolfram.com/LeastSquaresFittingExponential.html
4. [Benz, J. P. *et al.* Identification and characterization of a galacturonic acid transporter from *Neurospora crassa* and its application for *Saccharomyces cerevisiae* fermentation processes. *Biotechnol. Biofuels* **7,** 20 (2014).](http://paperpile.com/b/Rc0Kzu/KSS3)
5. Kwolek-Mirek, M & Zadrag-Tecza, R. Comparison of methods used for assessing the viability and vitality of yeast cells. **14**, 1068-79 (2014).
6. Wei, N, *et al*. Enhanced biofuel production through coupled acetic acid and xylose consumption by engineered yeast. **4**, 2580 (2013).
