# Supplementary material for: Engineering Saccharomyces cerevisiae for co-utilization of d-galacturonic acid and d-glucose from citrus peel waste
Source: Nat Commun. 2018 Nov 29;9:5059. doi: 10.1038/s41467-018-07589-w (PMC6265301; doi:10.1038/s41467-018-07589-w)
Supplement: Supplementary file 2 — Description of Additional Supplementary Files [file 41467_2018_7589_MOESM2_ESM.pdf]

Description of Additional Supplementary Files

Supplementary Data 1

Description: Supplementary sequence information for fungal D-galacturonic acid utilization pathway enzymes and GAT-1 homologues sequence codon optimized for expression in *S. cerevisiae* using the IDT codon optimization tool https://www.idtdna.com/CodonOpt and synthesized by IDT.

Supplementary Data 2

Description: Sequence information for promoters, terminators and spacers used for expression cassettes in *S. cerevisiae* as described in Lee, M.E et al.

Supplementary Data 3

Description: Primers for cloning transporters in *Neurospora* complementation strains.
